# Characterisation of a primary ciliary dyskinesia model generated from *BMI1*-transduced basal epithelial cells

**DOI:** 10.1242/jcs.263886

**Published:** 2025-10-31

**Authors:** Melis T. Dalbay, Eriomina Shahaj, Ileana Guerrini, Dani Do Hyang Lee, Anna Straatman-Iwanowska, Hannah M. Mitchison, Deborah L. Baines, Robert A. Hirst, Claire Hogg, Christopher O'Callaghan, Stephen L. Hart

**Affiliations:** ^1^ Department of Genetics and Genomic Medicine, UCL Great Ormond Street Institute of Child Health, University College London, London WC1N 1EH, UK; ^2^ Department of Infection, Immunity & Inflammation, UCL Great Ormond Street Institute of Child Health, University College London, London WC1N 1EH, UK; ^3^ Centre for Primary Ciliary Dyskinesia Diagnosis and Research, Department of Respiratory Sciences, University of Leicester, Leicester LE1 7HB, UK; ^4^ Institute for Infection and Immunity, St George's University, University of London, London EC1V 0HB, UK; ^5^ PCD Diagnostic Centre, Royal Brompton and Harefield NHS Trust, London SW3 6NP, UK; ^6^ Paediatric Respiratory Medicine, Imperial College London, London SW3 6NP, UK

**Keywords:** PCD, Airway cell culture models, ENaC

## Abstract

Primary ciliary dyskinesia (PCD) is a rare genetic respiratory disorder caused by a reduction in cilia number or cilia dysmotility. Cilia dysmotility leads to breathing difficulties, concurrent infections and severe lung damage if not treated, with no therapies currently available. Improved airway epithelial cell models that mimic the disease phenotype are required for development of new therapeutics, as current models have limited potential of self-renewal *in vitro*. Here, we describe a human PCD cell model created by lentiviral transduction of airway basal epithelial cells with the *BMI1* gene, a regulator of senescence. We report that the cells retain their proliferation and differentiation capacity for at least 19 passages and recapitulate the disease phenotype with immotile cilia lacking DNAH5 and other outer dynein arm proteins. Characterisation of the ion transport properties of these PCD cells grown at an air–liquid interface showed lower activity of the Na^+^ channel ENaC and enhanced CFTR activity compared to non-PCD cells, which might be linked to ciliary immotility. Our study provides a robust PCD model for therapeutic studies, opening new avenues to investigate the molecular mechanisms of this disease.

## Teaser

**Summary:** We developed a robust human PCD airway cell model via *BMI1* transduction, retaining proliferation, recapitulating immotile cilia and revealing altered ion transport, enabling therapeutic and mechanistic studies.

## INTRODUCTION

Primary ciliary dyskinesia (PCD) is an autosomal recessive genetic disorder, estimated to affect 1 in 10,000 live births, caused by variants in genes encoding proteins governing the multiciliogenesis programme that lead to ciliary dysmotility (Legendre et al., 2021). Motile cilia are small hair-like organelles that protrude from the surface of epithelial cells lining the airways, where they beat continuously to clear mucus and trapped debris and pathogens. While PCD manifests as sinusitis and rhinitis in the upper airways, cilia motility defects lead to a more severe condition in the lower airways, characterised by respiratory mucus entrapment and chronic bacterial infections leading to bronchitis and pneumonia. This results in breathing difficulties and progression to scarred, enlarged airways (bronchiectasis) and increasing deterioration of lung function (Lucas et al., 2014). There is currently no cure for PCD, just symptomatic treatment.

Motile cilia have an internal scaffold structure called the axoneme, which is formed of nine microtubule doublets surrounding (in most motile cilia) a central pair of microtubules (the ‘9+2’ array) (Satir and Christensen, 2007). Cilia contain hundreds of proteins, including dynein motor proteins that drive ciliary motility through ATP hydrolysis, by sliding along the outer microtubules (Brokaw and Kamiya, 1987; Burgess et al., 2003). Outer dynein arms (ODAs) regulate ciliary beat frequency (CBF), whereas inner dynein arms (IDAs) and nexin–dynein regulatory complexes link adjacent microtubules, regulating the axonemal waveform and beating pattern (Awata et al., 2015; Heuser et al., 2009). Radial spokes protrude from the peripheral microtubules towards the central pair, forming a radial scaffold that also helps mediate ciliary motility through mechanochemical transduction (Zhu et al., 2017). So far, variants in more than 50 different genes have been associated with PCD, affecting many structural elements of the axoneme (Legendre et al., 2021), with variants in the ODA heavy chain gene *DNAH5* being responsible for at least a third of north European PCD cases (Fassad et al., 2020; Hornef et al., 2006).

Research into therapies for PCD is hampered by the lack of robust models that recapitulate the disease phenotype. *In vivo*, PCD knockout mouse models with *Dnah5* variants usually die *in utero* or postnatally due to *situs inversus*-related cardiac conditions or hydrocephalus. Furthermore, these models fail to replicate respiratory phenotypes of PCD, such as bronchiectasis (Ibanez-Tallon et al., 2002; Ostrowski et al., 2010; Yin et al., 2019; Lee and Ostrowski, 2021). A mouse model of PCD associated with variants in another ODA heavy chain, *Dnah11*, was reported to display immotile tracheal cilia with normal ultrastructure and reduced sperm motility, accompanied by gross rhinitis, sinusitis and otitis media (Lucas et al., 2012), although no lower airway disease was reported and there are few subsequent reports using this model. A conditional transgenic model of PCD was generated in 6–11-week-old mice in which administration of tamoxifen induced *Cre* recombinase expression, excising the axonemal gene *Dnaic1* (also known as *Dnai1*). This approach avoided the development of hydrocephalus or cardiac conditions and led to symptoms of mucociliary defects in the lung; however, due to the slow turnover of ciliated cells in the airway, mice took up to 6 months to develop PCD symptoms, which included chronic rhinosinusitis (Ostrowski et al., 2010). Other murine models of PCD in which *Rsph1* is deleted develop respiratory symptoms characteristic of PCD, but milder than in other PCD mice and with less severe hydrocephalus, although less than a third of mice survive to adulthood (Yin et al., 2019). A recent mouse model study investigated effects of different variants in *Dnaaf5* in a wide range of tissues, reporting occurrence of differential phenotypes in different tissues, with no lung disorder presented in the lower airway, unlike the phenotype observed in people with PCD (Horani and Ferkol, 2018).

Cultures of human primary basal epithelial cells at an air–liquid interface (ALI) display differentiation and ciliogenesis, producing a pseudostratified epithelium that closely mimics the *in vivo* epithelium, and thus provide a robust, versatile model for studying PCD biology and potential therapies (Hirst et al., 2010). However, a significant drawback in the use of this model is that, due to senescence, the expansion of primary basal cell cultures is limited to two or three passages, limiting their long-term use and requiring repeated acquisition of fresh donor samples (Lechner et al., 1982). We have previously shown that transduction of basal epithelial cells with *BMI1*, which encodes a Polycomb RING finger protein, delays the onset of senescence for at least 20 passages while basal cells retain their capability to differentiate (Munye et al., 2017). *BMI1-*transduced basal cells from the bronchial epithelium of cystic fibrosis donors (CFBE cells) display characteristic ion transport and fluid homeostasis defects in ALI culture and have been used to assess physiological correction with an epithelial sodium channel (ENaC)-targeted small-interfering RNA (siRNA) therapy (Tagalakis et al., 2018).

Here, we report the development and characterisation of human PCD cell models, generated by *BMI1* transduction of bronchial and nasal primary basal epithelial cells from PCD donors biallelic for *DNAH5* variants. *BMI1*-transduced basal cells from both healthy controls and *DNAH5* variant donors were expanded in culture at least up to eighteen passages while retaining their ability to differentiate into a pseudostratified, ciliated epithelium. The epithelia derived from *DNAH5* variant donors displayed dysmotile cilia that lacked DNAH5 and other ODA proteins, thus recapitulating key features of PCD. In contrast, *BMI1-*transduced basal cells from the bronchial epithelium of healthy individuals displayed abundant ciliation with normal ciliary motility. In characterising these PCD models, the ciliary localisation of axonemal proteins – including DNAH5, DNAI1, RSPH1, CCDC39 and CCDC40 – was evaluated and the cilia ultrastructure was assessed using transmission electron microscopy (TEM). In addition, preliminary studies were performed using the *BMI1*-transduced PCD cells to investigate the expression and activity of ENaC and CFTR, which are ion transport channels that regulate homeostasis in the periciliary liquid layer and play an important role in mucociliary clearance.

## RESULTS

### Expansion and differentiation of *BMI1*-transduced PCD basal cells

**Fig. 1.**
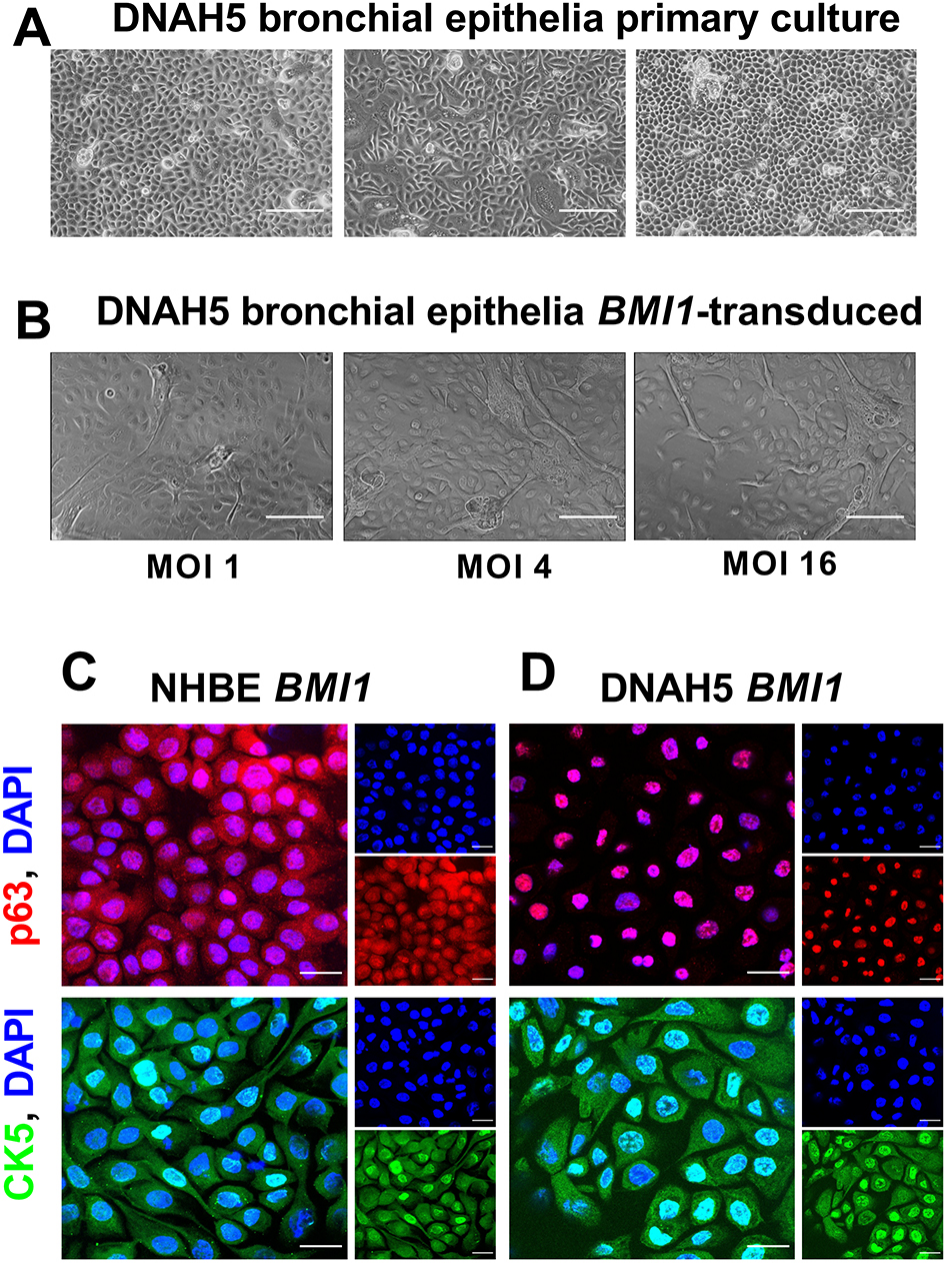
**Primary cell culture and lentiviral transduction of DNAH5-1 PCD bronchial epithelia.** (A) Primary bronchial airway epithelial cells from PCD donor DNAH5-1 at day 7 post seeding (three different wells). (B) DNAH5-1 cells after *BMI1* transduction (p2 cultures) at three different MOIs. Scale bars: 100 µm. (C,D) Overlay of confocal images showing nuclear expression of p63 (red) and nucleocytoplasmic expression of CK5 (green) basal airway stem cell marker in *BMI1*-transduced NHBE cells (p2,9) (C) and in *BMI1*-transduced cells from PCD donor DNAH5-1 (p1,10) (D). Nuclei were stained blue using DAPI. Scale bars: 20 µm. Images in C and D are representative of three experiments.

Nasal and bronchial brushing samples were obtained from two PCD donors, both biallelic for *DNAH5* variants (Table 1). Then, primary basal cells derived from donor 1 (DNAH5-1) were cultured in 12-well plates with growth medium for basal cell expansion. Cells reached 90% confluency in 7 days and displayed a characteristic cobblestone epithelium phenotype (Fig. 1A). Cells were then transferred to larger wells in 6-well plates at passage 1 (p1) and, 24 h post-seeding, were transduced with a lentiviral vector encoding *BMI1* at multiplicities of infection (MOIs) of 1, 4 and 16. Cells were maintained in the wells for the next 7 days then transferred to T75 flasks (passage 2, p2) and were further expanded in growth medium until 90% confluent. *BMI1*-transduced basal cells at all MOIs displayed characteristic basal epithelial cell morphology like their non-transduced primary cell counterparts, as shown at p2 for donor 1 in Fig. 1B. Cells obtained from nasal brushing of PCD donor 2 (DNAH5-2) displayed similar behaviour and appearance compared to the cells derived from the bronchial region of donor 1, DNAH5-1 (data not shown). The cells transduced at a MOI of 16 were selected for subsequent experiments based on growth rate studies conducted in *BMI1*-transduced normal human bronchial epithelial (NHBE) cells, where the MOI 16 cultures showed a constant growth rate for at least 85 days, whereas non-transduced primary cells grew at a slower rate, which declined from day 10 (Fig. S1). Immunofluorescence staining was then performed to assess the expression of the airway basal epithelial cell markers p63 (also known as TP63), a transcription factor required for epithelial progenitor cell renewal (Melino et al., 2015), and cytokeratin-5 (CK5, also known as KRT5), a filament protein characteristic of progenitor cells (Voynow et al., 2005). These markers were detected in *BMI1*-transduced NHBE and DNAH5-1 basal cells prior to differentiation in ALI culture (Fig. 1C,D). Positive staining for p63 and CK5, which are both characteristic of progenitor cells, suggests that *BMI1*-transduced cells retain their pluripotency.

**Table 1.** DNAH5 variants from PCD donors

Donor	Age at sampling	Sex	Allele 1 cDNA, protein	Allele 1 dbSNP ID	Allele 2 cDNA, protein	Allele 2 dbSNP ID	Tissue source
DNAH5-1	4 years	Male	c.10825C>T, p.(Gln3609Ter)	rs749980719	c.3466del, p.(Ile1156fs)	rs775866092	Bronchoscopy
DNAH5-2	8 years	Male	c.13458dup, p.(Asn4487Ter)	rs775696136	c.13486C>T, p.(Arg4496Ter)	rs200901816	Nasal brushing

### Characterisation of ALI cultures of *BMI1*-transduced PCD airway epithelial cells

**Fig. 2.**
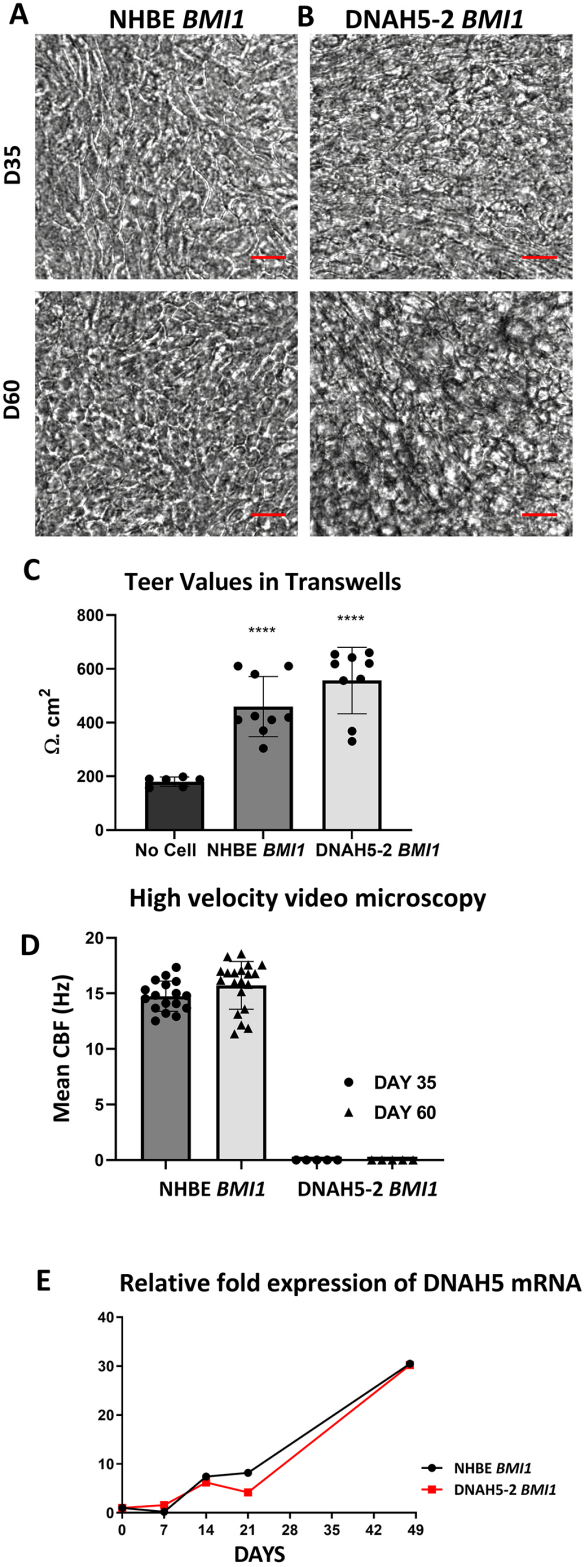
**Analysis of *BMI1*-transduced DNAH5-2 PCD airway epithelial cilia function.** (A) *BMI1*-transduced NHBE cells (p2,12) and (B) *BMI1*-transduced basal cells from donor DNAH5-2 (p1,12) grown on Transwell membranes at day 35 (D35) and day 60 (D60) of ALI culture. Images are representative of *n*=5 fields of view. Scale bars: 20 µm. (C) Epithelial cell resistances of *BMI1*-transduced NHBE and DNAH5-2 cells at day 30 of ALI culture were similar, with no statistically significant difference (two-tailed Mann–Whitney *U* test, ****P<0.001; *n*=9; mean±s.d.). (D) High-velocity video microscopy recordings show 14.74 Hz and 15.72 Hz CBF for *BMI1*-transduced NHBE cells at day 35 and day 60, respectively, indicating functional cilia motility, whereas *BMI1*-transduced DNAH5-2 cells display immotile cilia (*n*=17 and *n*=20 fields of view for NHBE cells at day 35 and day 60, respectively; *n*=5 fields of view for DNAH5-2 cells; mean±s.d.). (E) *DNAH5* mRNA expression in both *BMI1*-transduced NHBE cells and *BMI1*-transduced DNAH5 cells increased up to 30-fold over the course of 49 days of differentiation in ALI culture (mean of *n*=3 per time point).

ALI cultures were established from *BMI1*-transduced DNAH5-2 and NHBE basal cells, both at passage 12, and assessed for their epithelial phenotype. Both the NHBE cells and DNAH5-2 cells displayed an intact epithelial sheet on transwell membranes at day 35 post air-lift in ALI culture, therefore subsequent experiments were performed at this time point (Fig. 2A,B), although the epithelium maintained its differentiated appearance for at least 60 days of ALI culture (Fig. 2A,B). Transepithelial resistance (TEER) values were similar for both DNAH5-2 and NHBE ALI cultures, indicating the development of a functional epithelial barrier (Fig. 2C).

### ALI culture imaging and analysis for ciliary beat frequency by high-velocity video microscopy

Cilia of *BMI1*-transduced NHBE cells displayed a CBF of ∼15 Hz at day 35 and day 60 of ALI culture, which is in the normal range (Smith et al., 2012), and a normal ciliary beat pattern (Fig. 2D; Movies 1–4). The cilia in *BMI1-*transduced DNAH5-2 cells, however, were mostly static, consistent with the phenotype resulting from the lack of ODAs (Fig. 2D; Movies 5–8).

Expression of *DNAH5* was determined by reverse transcription-quantitative PCR (qRT-PCR) analysis of total cell mRNA from ALI cultures at 7 day intervals up to day 21 and at day 49. *DNAH5* expression in ALI cultures of both DNAH5-2 and NHBE cells increased from day 7 of ALI until day 49 for both cell lines, with no significant difference in the increase between them (Fig. 2E). Thus, *BMI1* transduction had no impact on the pattern of increasing *DNAH5* expression and associated ciliogenesis (Loges et al., 2018).

### Characterisation of ciliary proteins in PCD airway epithelial cells

**Fig. 3.**
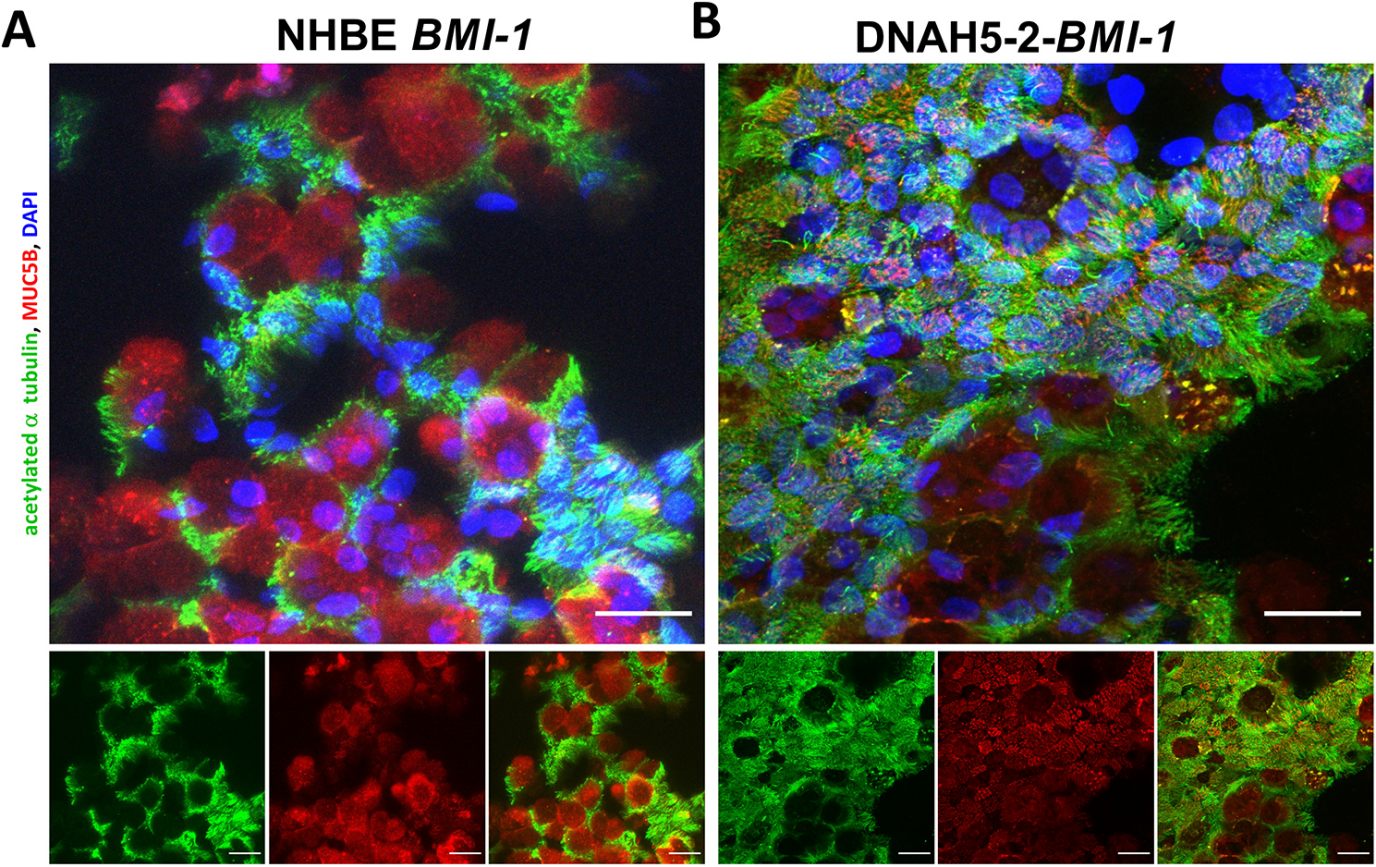
**Differentiation of *BMI1*-transduced *DNAH5-2* PCD airway progenitor cells.** Three-dimensional reconstructions of confocal images showing staining of acetylated α tubulin (green), MUC5B (red) and nuclei (DAPI, blue), demonstrating differentiation of basal airway cells into ciliated, mucus-producing cells at day 30 of ALI culture. (A) *BMI1*-transduced NHBE cells (p2,9) and (B) *BMI1*-transduced DNAH5-2 cells (p1,10). Scale bars: 20 µm. Images are representative of three experiments

**Fig. 4.**
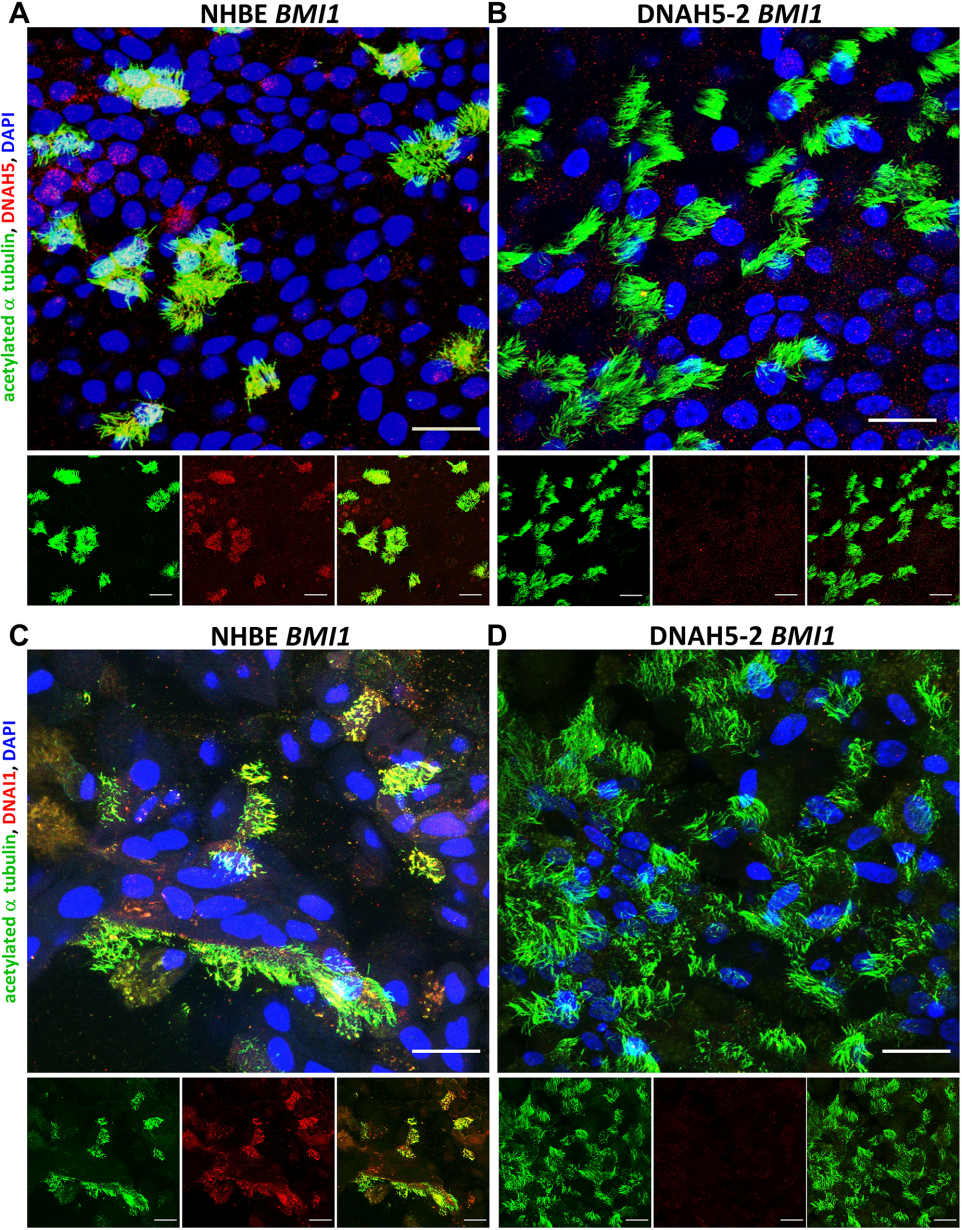
**Characterisation of DNAH5 and DNAI1 localisation in *BMI1*-transduced DNAH5-2 PCD airway cell cilia.** Three-dimensional reconstructions of confocal images showing ciliary localisation of acetylated α tubulin (green) and either DNAH5 (A,B; red) or DNAI1 (C,D; red) in differentiated airway epithelial cells, with DAPI (blue) staining of the nucleus. (A) DNAH5 was present in cilia in *BMI1*-transduced NHBE cells (p2,9) but (B) absent in the cilia of *BMI1*-transduced DNAH5-2 cells (p1,10). (C) DNAI1 was present in cilia in *BMI1*-transduced NHBE cells but (D) absent in in the cilia of *BMI1*-transduced DNAH5-2 cells at day 30 of differentiation. Scale bars: 20 µm. Images are representative of three experiments.

We next analysed the ALI cultures of *BMI1*-transduced PCD cells by immunofluorescence staining for evidence of differentiation; namely, ciliation and mucus production. Immunofluorescence images of ALI cultures of both NHBE and DNAH5-2 cells at day 30, acquired using confocal microscopy, displayed densely ciliated cultures, as detected by staining for acetylated α tubulin, as well as mucus production by secretory cells, detected by staining for the mucin MUC5B (Fig. 3A,B). Immunofluorescence staining was then performed to assess the ciliary localisation of DNAH5 and other proteins involved in cilia structure and motility. DNAH5 was detected in the motile cilia of *BMI1*-transduced NHBE cells (Fig. 4A) but, consistent with their genotype, was absent from the cilia of *BMI1*-transduced DNAH5-2 and DNAH5-1 cells (Fig. 4B; Fig. S2), suggesting impaired ciliary trafficking of the variant proteins.

Immunofluorescence analysis of another ODA protein, DNAI1, revealed its colocalisation with DNAH5 along the length of the cilia in NHBE cells and, as expected, its absence from cilia of DNAH5-2 and DNAH5-1 cells since the entire ODA is missing (Fig. 4C,D; Fig. S3) (Loges et al., 2018).

**Fig. 5.**
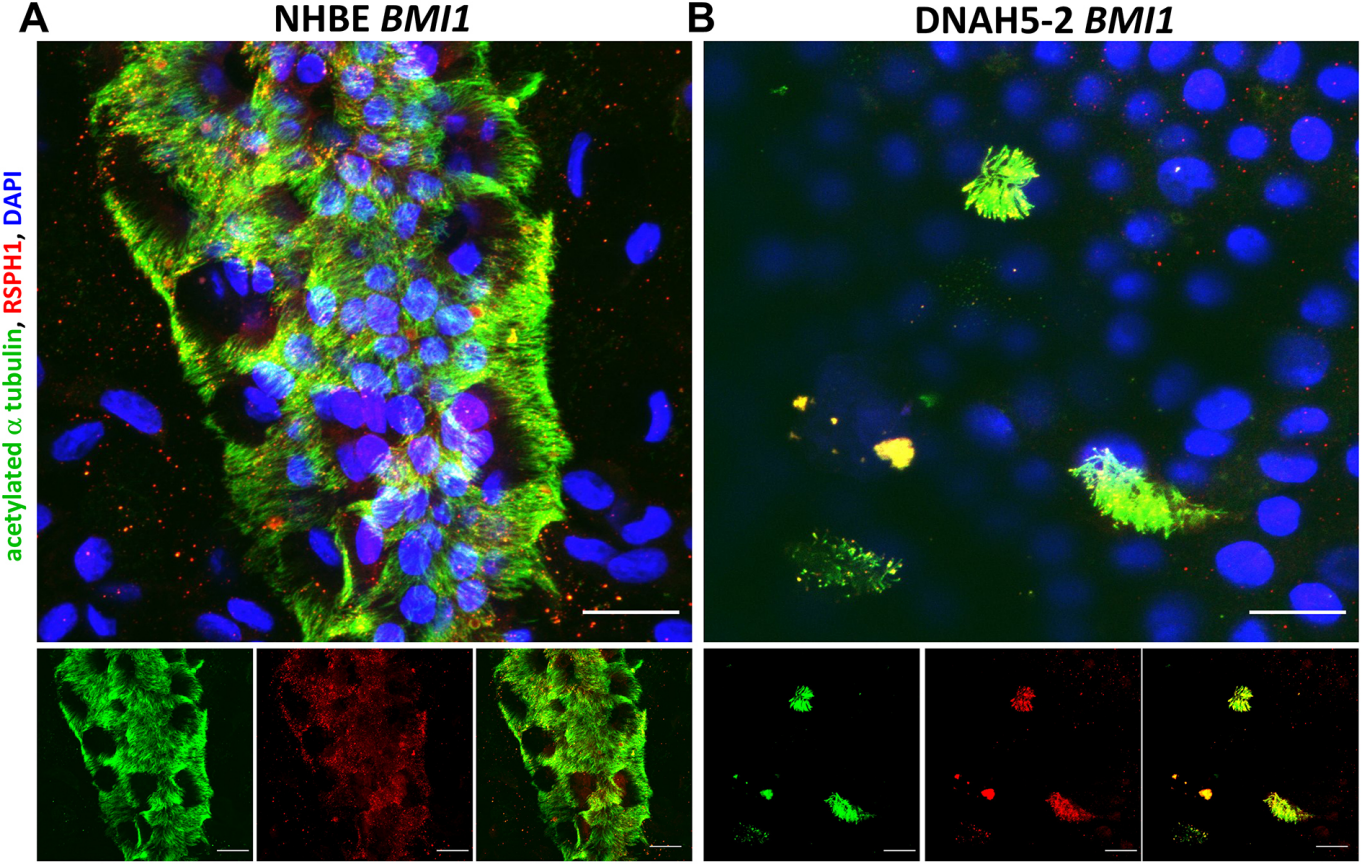
**Characterisation of RSPH1 localisation in *BMI1*-transduced DNAH5-2 PCD airway cell cilia.** Three-dimensional reconstructions of confocal images showing ciliary localisation of acetylated α tubulin (green) and RSPH1 (red) in differentiated airway epithelial cells, with DAPI (blue) staining of the nucleus. RSPH1 was present in cilia in both (A) *BMI1*-transduced NHBE cells (p2,9) and (B) *BMI1*-transduced DNAH5-2 cells (p1,10) at day 30 of differentiation. Scale bars: 20 µm. Images are representative of three experiments.

**Fig. 6.**
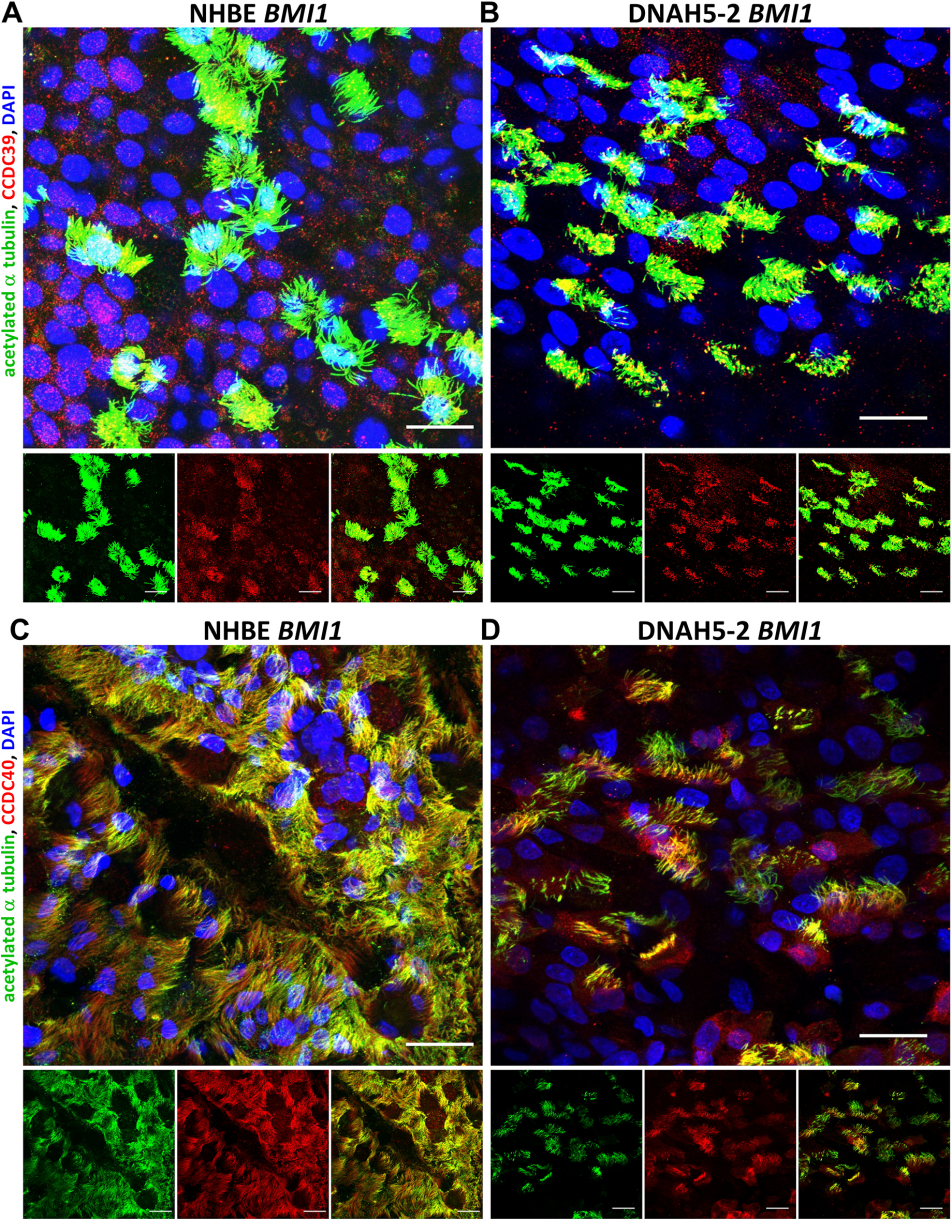
**Characterisation of CCDC39 and CCDC40 localisation in *BMI1*-transduced DNAH5-2 PCD airway cell cilia.** Three-dimensional reconstructions of confocal images showing ciliary localisation of acetylated α tubulin (green) and either CCDC39 (A,B; red) or CCDC40 (C,D; red), as well as DAPI (blue) staining of the nucleus, in differentiated airway epithelial cells. CCDC39 was shown to be present in cilia of both (A) *BMI1*-transduced NHBE cells (p2,9) and (B) *BMI1*-transduced PCD cells from donor DNAH5-2 (p1,10). CCDC40 was localised in cilia of both (C) *BMI1*-transduced NHBE cells and (D) *BMI1*-transduced DNAH5-2 cells at day 30 of differentiation. Scale bars: 20 µm. Images are representative of three experiments.

**Fig. 7.**
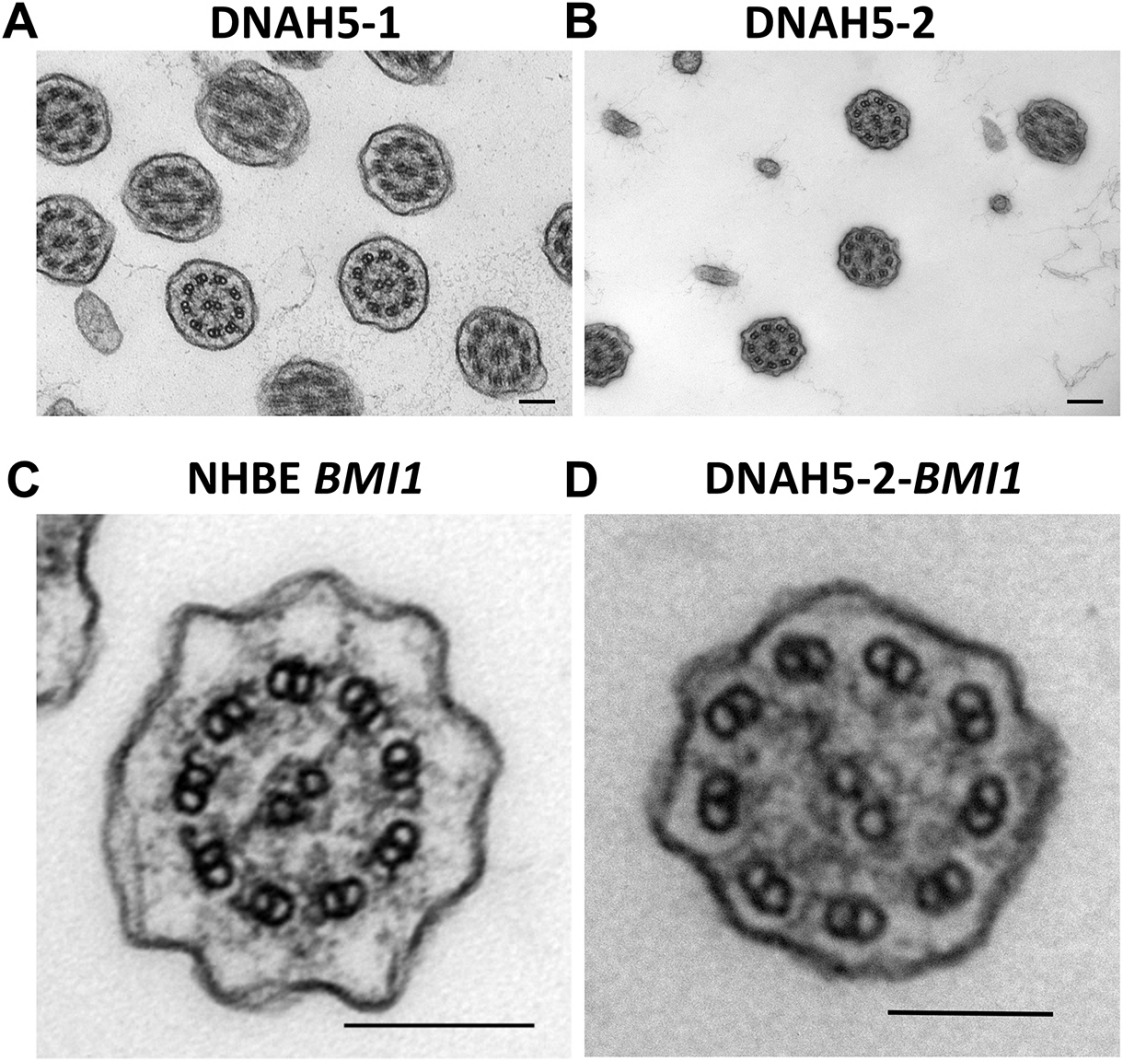
**TEM analysis of ODAs in *BMI1*-transduced *DNAH5* variant PCD airway cell cilia.** (A,B) Cross sections showing the cilia of primary cells before *BMI1* transduction from (A) donor DNAH5-1 and (B) donor DNAH5-2. TEM images show missing IDAs and ODAs. Scale bars: 100 nm in A, 200 nm in B. (C,D) Cross sections showing cilia of (C) a *BMI1*-transduced NHBE cell, with IDA and ODA present, and (D) a *BMI1*-transduced DNAH5-2 cell, with missing IDA and ODA. Scale bars: 100 nm. Images are representative of *n*=20 fields of view.

Other cilia structural proteins, including the radial spoke protein RSPH1 (Fig. 5A,B; Fig. S4) and the molecular ruler proteins CCDC39 and CCDC40 (Fig. 6; Figs S5 and S6), were all detected to be localised in *BMI1*-transduced NHBE, DNAH5-2 and DNAH5-1 cilia, indicating that they are unaffected by the loss of DNAH5, as expected for non-ODA structural proteins. The loss of ciliary ODAs in ALI cultures of both DNAH5-1 and DNAH5-2 cells was confirmed by TEM analysis of primary cell ALI cultures before *BMI1* transduction (Fig. 7A,B) and of DNAH5-2 cells after *BMI1* transduction, whereas ODA structures were normal in *BMI1*-transduced NHBE cells (Fig. 7C,D).

### Comparison of *BMI1*-transduced PCD cell characteristics to primary PCD cells

To investigate whether *BMI1*-transduced PCD airway epithelial cells retain their phenotype, capacity of growth and differentiation potential over the long term, we generated ALI cultures from *BMI1*-transduced basal cells at passage 1 (p1) and evaluated them at the later passages of p15 and p19 (DNAH5-1 cells at p1,15 and p1,19; here and below, passage numbers are denoted as the passage number at *BMI1* transduction followed by the passage number at which cells were utilised) and compared them against their primary cell counterparts. ALI cultures established from both p1,15 and p1,19 *BMI1*-transduced DNAH5-1 cells formed intact epithelial sheets on Transwells, retaining an epithelial cell morphology similar to that of primary DNAH5-1 cells of passage 3 (Fig. S7A). TEER values of *BMI1*-transduced p1,19 DNAH5-1 cells were slightly higher than those of the non-transduced cells (Fig. S7B), indicating that they retained the ability to form an intact epithelial barrier despite their high passage number, whereas CBFs for both were static, retaining the PCD phenotype (Fig. S7C).

TEM analysis of *BMI1*-transduced p1,19 DNAH5-1 cells grown in ALI cultures revealed cilia structures similar to the corresponding non-transduced primary cell cilia, with missing ODAs (Fig. S7D). In order to assess their proliferation potential over time, *BMI1*-transduced DNAH5-1 cells from different passage numbers (p1,8 and p1,12) were maintained in cell culture to 60 days and growth curves were plotted. Both p1,8 and p1,12 DNAH5-1 *BMI1*-transduced cells displayed similar growth rates, whereas their primary cell counterparts displayed growth arrest after three passages (Fig. S7E). These results indicate that *BMI1*-transduced PCD cells can retain their proliferation potential in the long term, exhibiting similar growth kinetics at later passages. Confocal analysis to assess the differentiation potential of *BMI1*-transduced DNAH5-1 late passage cells (p1,19) and non-transduced primary DNAH5-1 cells in ALI cultures showed that the *BMI1*-transduced cells of passage 19 were as capable of differentiation as the non-transduced cells, with both displaying a smaller proportion of secretory cells producing a similar level of MUC5AC, as measured across six random fields-of-view imaged, with a slightly higher percentage of ciliated fields-of-view measured in primary DNAH5-1 cultures than in the late passage cultures (Fig. S8).

### ENaC and CFTR expression and ion transport activity in PCD cells

**Fig. 8.**
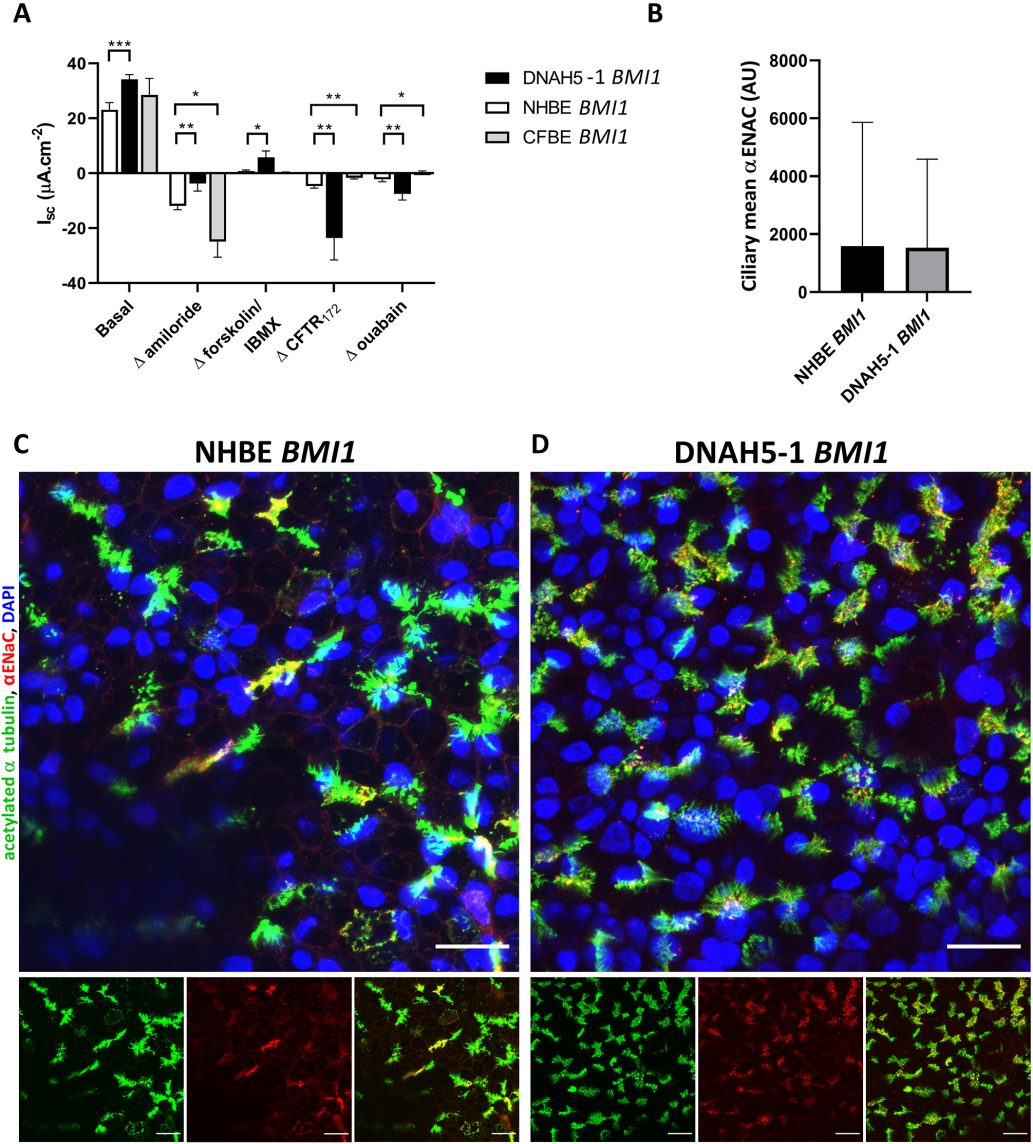
**Characterisation of ion transport in *BMI1*-transduced DNAH5-1 PCD airway cells and ciliary ENaC localisation.** (A) Spontaneous (basal) mean short circuit current (*I*_sc_) or change in *I*_sc_ (Δ*I*_sc_) in response to drug treatments inhibiting ENaC (Δ amiloride), activating CFTR (Δ forskolin/IBMX), inhibiting CFTR activity (Δ CFTR_172_) or inhibiting Na^+^/K^+^-ATPase (Δ ouabain) recorded for *BMI1*-transduced NHBE cells, *BMI1*-transduced DNAH5-1 cells (bronchial origin) and *BMI1*-transduced CFBE cells at day 30 of ALI culture. Inhibitory Δ*I*_sc_ are shown as negative changes and stimulatory Δ*I*_sc_ are shown as positive changes. Mean±s.d., *n*=3. *P<0.05, **P<0.01, ***P<0.001 (two-tailed Mann–Whitney *U* test). See Materials and Methods for details of drug treatments. (B) Mean ciliary αENaC of *BMI1*-transduced NHBE and DNAH5-1 cells from three-dimensional reconstructions of confocal images of ALI cultures at day 30, quantified using Imaris software. Mean±s.d., *n*≥5 fields of view, at least 10,000 ciliary measurement per sample. AU, arbitrary units. (C,D) Three-dimensional reconstructions of confocal images showing ciliary localisation of acetylated α tubulin (green) and αENaC (red) in (C) differentiated bronchial epithelial cells (*BMI1*-transduced NHBE cells; p2,17) and in (D) differentiated *BMI1*-transduced DNAH5-1 donor airway cells (p1,18) at 30 days of differentiation. Nuclei are stained using DAPI (blue). Scale bars: 20 µm.

The epithelial Na^+^ channel ENaC and the anion channel CFTR play important roles in regulating not just ion flow but also fluid flow across the epithelium, impacting on mucociliary clearance through their effects on the depth of the airway surface liquid (ASL), which comprises the mucus and periciliary liquid layers, and the viscosity of the mucus. Therefore, it was important to confirm these ion channels are expressed in *BMI1*-transduced PCD cells. Ion channel activities were measured as short-circuit currents (*I*_sc_) of ENaC and CFTR in *BMI1*-transduced PCD airway epithelial cells and were compared to *BMI1-*transduced NHBE and cystic fibrosis cells (CFBE cells)*.* Spontaneous (basal current) *I*_sc_ was first measured, which revealed differences in net ion transport between the bronchial PCD cells (DNAH5-1) and NHBE cells (Fig. 8A). The change in *I*_sc_ after application of amiloride to inhibit ENaC was lower in DNAH5-1 cells than in NHBE cells or CFBE cells, indicating less ENaC channel activity in the bronchial PCD cells (Fig. 8A), although western blot analysis suggested that the major ENaC subunit, αENaC (also known as SCNN1A), was expressed at slightly higher levels in the PCD cells relative to NHBE cells (Fig. S9). Amiloride induced a greater *I*_sc_ inhibition in CFBE cells than in NHBE cells, indicating higher ENaC activity in the CFBE cells that is characteristic of a cystic fibrosis epithelium (Fig. 8A) (Moore and Tarran, 2018). Thus, these data indicate that ENaC channel activity is lower in the ALI cultures of PCD bronchial epithelial cells. Immunofluorescence staining of *BMI1*-transduced NHBE and PCD bronchial airway epithelial cells showed that αENaC was distributed in the membrane along the length of the ciliary axonemes in both cell types in similar amounts (Fig. 8B–D), consistent with the western blot analysis (Fig. S9). Despite the similar ciliary levels of αENaC observed in NHBE and DNAH5-1 cells, total αENaC protein levels by western blot analysis were higher in both PCD cell lines (DNAH5-1 and DNAH5-2) than in NHBE cells and CFBE cells, with the latter exhibiting levels of total αENaC protein that were lower than those in NHBE cells despite their higher ENaC ion channel activity. Thus, the differences in ENaC activity observed in Ussing chamber analysis of ALI cultures are probably a consequence of regulatory control of ENaC rather than abundance of the protein or distribution of the channel. ENaC is subjected to a complex array of post-translational regulatory processes – including protease activation and CFTR inhibition, which has been studied in relation to cystic fibrosis (Mall, 2020; Matalon et al., 2015) – but there is little known of ENaC regulation in relation to ciliary motility in PCD (Wu et al., 2018).

ALI cultures of PCD bronchial epithelial cells displayed greater change in *I*_sc_ in response to treatment with IBMX and forskolin than the NHBE cells, indicating greater activation of CFTR, and the change in *I*_sc_ in response to the CFTR-specific inhibitor CFTR_inh_-172 was also greater in PCD bronchial epithelium (DNAH5-1) than in NHBE epithelium (Fig. 8A). CFBE epithelium, as expected, yielded little response to activation with IBMX and forskolin or to inhibition with CFTR_inh_-172, consistent with the absence of CFTR. In addition to higher CFTR ion channel activity in bronchial PCD cells, both PCD cell types in ALI cultures displayed higher levels of CFTR protein than the NHBE cells, with both mature (band C) and immature forms (band B) of CFTR present, suggesting the presence of functional CFTR protein (Fig. S9). Analysis of more donor samples is required to explore whether the increased activity and abundance of CFTR in PCD epithelium is a disease-specific phenotype. Finally, the inhibitory change in *I*_sc_ in response to ouabain was greater in DNAH5-1 cells than in NHBE or CFBE cells. As ouabain inhibits all ion transport processes in airway cells, these data indicate that ion transport activities other than those associated with ENaC and CFTR might be upregulated in PCD cells.

Further studies will be required with cells from a wider range of PCD donors, but the striking differences in ENaC and CFTR activity and protein levels of the *BMI1-*transduced bronchial PCD cells used in this study raise questions of whether this might be linked to impaired ciliary function. ENaC and CFTR play central roles in ASL homeostasis, and so we hypothesise that increased CFTR and reduced ENaC activity might enhance hydration of the lung epithelium, compensating partially for the loss of mucociliary clearance in PCD cells. Further studies will be required to explore this hypothesis, but the *BMI1-*transduced basal PCD cells provide a consistent reliable resource for mechanistic studies without having to resample the same donor.

## DISCUSSION

Cell culture models that mimic the phenotype of the PCD airway epithelium are useful for studying airway disease mechanisms, discovering new genetic components involved in the disease and developing new therapies. There is a shortage of convenient models of PCD that display phenotypes specific to the underlying genetic variants, and so we have developed and analysed cell models from two PCD donors with variants in the gene encoding DNAH5, a critical protein in the ODA, which we propose could be useful for research into PCD. Absence of DNAH5 in individuals with PCD leads to loss of the ODAs and ciliary dysmotility.

Basal epithelial cells, obtained from biopsies or airway brushings, are progenitors of the differentiated airway epithelia and can be differentiated in ALI cultures to mimic the human airway *in vitro*. However, primary basal cells are capable of expansion for only two or three passages before losing their differentiation capacity, and so different approaches to enable expansion of these cells, while retaining their differentiation capacity, have been explored. Maintaining basal cells in co-culture with irradiated mouse feeder fibroblast cells and with Rho-associated protein kinase (ROCK) inhibitor in the medium results in a high yield of cells in successive passages that differentiate upon air-lift in ALI cultures (Butler et al., 2016). However, reports suggest that this approach maintains differentiation efficiency only up to 11 passages and that the cells also display changes in morphology (Reynolds et al., 2016; Suprynowicz et al., 2012). Moreover, the co-culture system is labour intensive and time consuming and might cause concerns over xenogenicity for downstream studies (Llames et al., 2015). Airway epithelial cells, including those from PCD donors, have been generated from induced pluripotential stem cells (iPSCs) but the process is prolonged, taking several weeks, and laborious (Hawkins et al., 2021). Human bronchial epithelial cells immortalised by simian virus 40 (SV40) large T antigen transformation, such as the well-known 16HBE14o- cell line, can form stratified epithelia and tight junctions and are useful for ion transport studies, but fail to produce mucus or cilia (Gruenert et al., 1988), limiting their utility for studies of PCD. Bronchial epithelial cells immortalised with HPV E6/E7 in conjunction with human telomerase reverse transcriptase (hTERT) have karyotypic abnormalities and progressively lose cilia with increasing passage number (Zabner et al., 2003). To resolve the problem of viral oncogenes, a significant advance was made by transducing human bronchial epithelial cells with the mouse Polycomb RING finger oncogene *Bmi1* and hTERT, creating cell lines of normal karyotype and no nuclear abnormalities; however, differentiation was defective, with mostly mucus-producing cells and only few ciliated cells (Fulcher et al., 2009). We have reported previously that transduction of human primary bronchial epithelial cells from healthy donors (Munye et al., 2017) and individuals with cystic fibrosis (Tagalakis et al., 2018) using human *BMI1* alone resolves these problems, establishing cell lines with normal karyotype, electrophysiological properties, mucociliary functionality, and sustained proliferative and differentiation properties for up to 25 passages (Munye et al., 2017).

In this study, *BMI1*-transduced *DNAH5* variant PCD cell models were established from both bronchoscopy and nasal brushings, demonstrating extended capacity for replication and differentiation. *BMI1*-transduced cells expressed the basal cell markers p63 and CK5, indicating they retained their pluripotency. Upon seeding basal cells on porous inserts with differentiation medium at the basal side and air exposure at the apical side, pseudostratified airway epithelia were generated, with ciliated and mucus-producing cells, even after repeated passaging of the basal cells. *BMI1-*transduced PCD basal cells could be expanded for at least 19 passages while retaining their differentiation capacity and could be returned to culture after storage in liquid nitrogen. In this study we have shown that *BMI1* transduction of PCD basal epithelial cells provides a robust, donor-derived cell model for long-term expansion and differentiation in ALI cultures.

The *BMI1*-transduced *DNAH5* variant cells in ALI culture accurately recapitulate the PCD phenotype, with static and dysmotile cilia, whereas cilia in *BMI1*-transduced NHBE airway cells derived from healthy donors display normal ciliary motility. Immunostaining showed that DNAH5 and its associated intermediate chain partner (DNAI1) were absent from the cilia of DNAH5-defective cells. In contrast, non-ODA proteins such as the nexins CCDC39 and CCDC40, as well as the radial spoke protein RSPH1, were detected in the cilia of these cells (Enuka et al., 2012; Loges et al., 2018; Fliegauf et al., 2005). *DNAH5* mRNA transcripts were present in the PCD cells, and their levels increased during ciliogenesis in ALI culture, as was observed in NHBE cells. The presence of *DNAH5* mRNA in differentiating DNAH5-2 cells that contain biallelic nonsense variants suggests that *DNAH5* transcripts were not fully depleted by nonsense-mediated decay (NMD). Transcripts with premature termination codons (PTC) can be resistant to NMD, enabling translation of truncated products (Kim et al., 2017). The absence of DNAH5 protein in the cilia despite the presence of *DNAH5* mRNA transcripts in DNAH5-2 cells suggests the inability of the truncated protein to be trafficked into the cilium, as was the case for DNAH5-1 cells, which contained one allelic nonsense variant and one missense variant (Table 1).

ENaC was detected in the membrane of the motile cilia of both *DNAH5* variant cells and NHBE cells, where it is ideally placed to regulate the volume of the ASL, which bathes the cilia (Hanukoglu and Hanukoglu, 2016). Analysis of the electrophysiological properties of the bronchial *DNAH5* variant *BMI1*-transduced epithelium at day 30 of ALI culture revealed significantly decreased responses to amiloride compared to the responses of ALI cultures of NHBE cells and CFBE cells, indicating decreased ENaC activity in the PCD ALI culture. The PCD epithelium from ALI cultures, however, appeared to contain amounts of ciliary αENaC protein similar to those in NHBE ALI cultures, suggesting that a regulatory process might have led to reduced ENaC activity. ENaC is regulated by proteases (Myerburg et al., 2010), but there is growing evidence that ENaC is activated by mechanical forces such as hydrostatic pressure or membrane stretching, as well as shear stress caused by fluid flow, in several organs and cell types, including oocytes, endothelium and kidney (Shi et al., 2013), and evidence of fluid-flow shear stress activation of ENaC in lung cells (Fronius et al., 2010). So, the lack of ciliary movement and loss of fluid flow might be playing roles in regulating ENaC in ALI cultures of the *BMI1*-transduced PCD cells. CFTR is also thought to play a role in regulating ENaC activity (Rooj et al., 2021), and interestingly, CFTR activity was elevated in ALI cultures of our bronchial PCD line compared to the activity in NHBE cells. These studies were performed with only one PCD cell type, and so it is not possible to draw firm conclusions on ion transport properties of the PCD phenotype, but they do suggest this might be an area that requires further investigation with larger sample numbers (Tosoni et al., 2016).

Loss of ENaC combined with enhanced CFTR activity would be expected to increase ASL volume, although a previous study has reported that PCD cells have a normal ASL depth (Tarran et al., 2006). However, those experiments focused on measuring the ability of the PCD cells to restore ASL volume upon addition of PBS and did not examine ENaC activity levels during the process. Interestingly, a clinical trial has been performed with the nebulised ENaC inhibitor VX-371 (also known as P-1037) and hypertonic saline, with the aim of clearing mucus obstructions by increasing hydration in the airways (clinicaltrials.gov; identifier NCT02871778). Our studies suggest that although αENaC protein levels are elevated in PCD, channel activity is not; this might be the result of regulation of ENaC activity, which can help to increase airway surface fluid and enhance mucus clearance – a process that is impaired in PCD. The *BMI1*-transduced PCD, NHBE and CFBE cells described here will enable further studies of the inter-relationship of ion and fluid homeostasis in the airways in relation to ciliary activity.

The *BMI1*-transduced basal cells from PCD donors lacking DNAH5 described here represent a useful resource for the study of PCD biology and are capable of long-term culture and differentiation. Biobanking of PCD cells offers a potentially useful resource for studies into PCD biology and therapies, and the *BMI1* transduction process could enhance the utility of stored cells, as the resulting cultures reliably recapitulate the disease phenotype *in vitro*. Recent studies have identified differential gene expression profiles in airway cells of PCD donors with *DNAH5* variants that have been suggested to potentially provide a compensatory mechanism for loss of DNAH5 function, which could be investigated using our model (Koenitzer et al., 2024; Yang et al., 2022). The method is simple and reproducible in the culture of donor-specific airway basal cells, rendering them a suitable system for use in the development of novel therapies ranging from read-through drugs to genetic therapies (Paff et al., 2021).

## MATERIALS AND METHODS

### Subjects

All donor samples were obtained with the individual's consent under local and national ethical approvals (London - Bloomsbury Research Ethics Committee, 08/H0713/82; Northwest - Liverpool Central Research Ethics Committee, 14/NW/0128), and in accordance with the principles expressed in the Declaration of Helsinki. Donor genotypes were confirmed previously, with variant nomenclature according to *DNAH5* transcript NM_001369.3. Airway epithelial cells harbouring predicted loss-of-function *DNAH5* variants were collected from two PCD donors, one carrying two nonsense variants and the other carrying a frameshift variant and a nonsense variant (Table 1). All variant alleles are known to be pathogenic (www.ncbi.nlm.nih.gov/clinvar/). Normal human bronchial epithelial cells (NHBE) were obtained from The Royal Institution for the Advancement of Learning, McGill University, Montreal, Canada at passage 1. CFBE cells, homozygous for the common ΔF508 variant, were obtained from Epithelix (Geneva, Switzerland).

### *BMI1* transduction of primary airway epithelial cells

Primary basal cells from bronchial or nasal brushings of the two PCD donors were expanded on 12-well plates in PneumaCult-Ex^TM^ growth medium (StemCell Technologies, Cambridge, UK). Cells were harvested and seeded at 1×10^5^ cells/well in 6-well plates in PneumaCult-Ex^TM^ growth medium (StemCell Technologies, Cambridge, UK). The next day, cells were transduced using LV-BMI PURO virus (Munye et al., 2017) in Opti-MEM (Thermo Fisher Scientific, UK) at multiplicities of infection (MOI) of 1, 4 and 16. The *BMI1* plasmid map is shown in Fig. S10. Lentivirus preparation and titration protocol were performed as described previously (Maeshima et al., 2024). PneumaCult-Ex^TM^ growth medium (StemCell Technologies, UK) was added to the cells the following day, then the medium was changed every 2 days until the cells reached 90% confluence, typically at day 7. Cells were harvested and transferred into T75 flasks pre-coated with 10% collagen I (ColI; PureCol^®^ Bovine Collagen Solution, Type I, Advanced BioMatrix, San Diego, CA, USA) for further expansion in PneumaCult-Ex^TM^ growth medium (StemCell Technologies, Cambridge, UK). All tissue culture plasticware was coated with ColI prior to seeding the cells.

### Air–liquid interface cultures

A million cells were plated per 12 mm-diameter well on collagen-coated Transwell or Snapwell inserts [Polyester (PET) Membrane Transwell-Clear Inserts, Corning, Corning Inc. Life Sciences, Flintshire, UK]. They were grown up to 5 days in PneumaCult Ex^TM^ growth medium (StemCell Technologies, Cambridge, UK) on both apical and basal sides. After 5 days the apical medium was removed and growth medium was changed to ALI culture medium (PneumaCult ALI^TM^, StemCell Technologies, Cambridge, UK) for differentiation. For this, the medium was changed every 2 days, and cells were analysed after day 30 of differentiation.

### Immunofluorescence staining and confocal analysis

Cells on Transwell membranes were fixed in 4% paraformaldehyde (PFA) for 10 min, washed in phosphate-buffered saline (PBS; Sigma-Aldrich, Gillingham, UK), permeabilised with Triton-X100 (0.2%; Bio-Rad, Hertfordshire, UK) in PBS for 10 min, then washed with PBS three times for 5 min per wash. Membranes were blocked with 5% goat serum (Abcam, Cambridge, UK) and 1 mg/ml bovine serum albumin (BSA) in PBS for 30 min at room temperature, followed by overnight incubation with primary antibodies at 4°C. Samples were washed three times with BSA (0.1 mg/ml) in PBS for 5 min per wash, followed by incubation with secondary antibodies for 1 h at room temperature. After three more wash steps with BSA (0.1 mg/ml) in PBS, nuclear staining was performed using DAPI solution (Thermo Fisher Scientific, Hemel Hempstead UK). Transwell membranes were cut out of holders using a scalpel and transferred onto slides with the apical surface facing up and mounted using *n*-propyl gallate (0.5% *n*-propyl gallate in glycerol, 0.1 M Tris-HCl pH 8.0).

Primary antibodies used were: mouse anti-acetylated α tubulin (TUBAIA ace-40Lys; 66 200-1 Ig) from Proteintech, Manchester, UK; rabbit anti-DNAH5 (HPA037470, lot B1144222) and rabbit anti-CCDC39 (HPA035364, lot A106506) from Atlas Antibodies, Bromma, Sweden; rabbit anti-CCDC40 (HPA022974; diluted 1:1000), rabbit anti-RSPH1 (HPA017382; diluted 1:100), rabbit anti-DNAI1 (HPA028305; diluted 1:200) and rabbit anti-MUC5B (HPA008246) from Sigma-Aldrich, Gillingham, UK; mouse anti-cytokeratin 5 (anti-CK5; ab17130) and rabbit anti-p63 (ab53039) from Abcam, Cambridge, UK; and rabbit anti-αENaC (SCNN1A; PA1-920A; Thermo Fisher Scientific, Hemel Hempstead, UK). Unless stated otherwise, primary antibodies were diluted 1:500 for use. Secondary antibodies were Alexa Fluor 488 F(ab′)2 fragment of goat anti-mouse IgG (H1L) and Alexa Fluor 633 goat anti-rabbit IgG (H1L) (both used at 1:1000 dilution; both from Molecular Probes, Life Technologies, Paisley, UK). For primary cell immunofluorescence, the following conjugated antibodies were used: anti-acetylated α tubulin (6-11B-1) Alexa Fluor 647 conjugated (sc-23950, lot K0724) from Santa Cruz Biotechnology, Dallas, USA; and mouse anti-MUC5AC Alexa Fluor 488 conjugated (NBP2-32732, lot D185610) from Biotechne, MN, USA. Samples were imaged using a Zeiss LSM710 confocal microscope (Zeiss, Cambridge, UK) with a 63× objective. Samples were imaged using ZEN blue software (Zeiss), with multiple *z*-sections taken through the thickness of the cell at 500 nm intervals. Maximum projections of *z*-stacks were recorded and analysed using Fiji ImageJ software (https://imagej.net/software/fiji/), and ciliary ENaC was quantified using Imaris software (Oxford Instruments, Oxford, UK), detecting each ciliary axoneme in *z*-stacks and automatically drawing boundaries around the cilia, with a diameter <0.3 µm set to detect each axonemal microtubule.

### qRT-PCR

Total cell RNA was extracted from cells using an RNeasy mini kit (Qiagen, Crawley, UK). Complementary DNA (cDNA) synthesis and quantitative PCR were performed from RNA samples using Sensi Fast SYBR Hi-ROX One step kit (Bioline, London, UK) in a Bio-Rad CFX96 thermal cycler (Bio-Rad, Watford, UK). *DNAH5* (Hs00292485-m1) expression and *β-actin* (Hs01060665-g1) reference gene expression were quantified using Taqman primers and probes (Applied Biosystems, Warrington, Cheshire, UK). PCR was performed at 45°C for 20 min, 95°C for 2 min followed by 40 cycles at 95^o^C for 15 s and 60°C for 1 min. Relative gene expression levels were determined using the delta-delta Ct (2^−ΔΔCt^) method (Livak and Schmittgen, 2001).

### Ciliary motility analysis

The CBF, ciliary beat pattern and other morphological features of the cilia were analysed by high-speed video microscopy as described previously (Thomas et al., 2010). Briefly, cells were suspended in HEPES (20 mM)-buffered medium 199 (GIBCO, ThermoFisher Scientific, Hemel Hempstead, UK) containing penicillin (50 µg/ml), streptomycin (50 µg/ml) and Fungizone (1 µg/ml; GIBCO, ThermoFisher Scientific, Hemel Hempstead, UK). Strips of ciliated epithelium at 37°C were imaged using a 100× objective and digitally recorded using a high-speed camera (EMMA, Nikon Ti-E). Ciliary activity was recorded at a rate of 500 frames per second (fps) and played back at reduced frame rates for ciliary beat pattern and CBF analysis. CBF was calculated using the following equation: CBF= (500/number of frames for 10 ciliary beats)×10, and a mean CBF was reported from a minimum of six measurements on independent ciliated strips. Cilia movies were taken from ten strips of ciliated epithelium at 500 fps. The normal range of CBF was 10–14 Hz, whereas immotile cilia had a CBF of 0 Hz. The ciliary beat pattern was defined as normal, dyskinetic or immotile as viewed from the side profile. Dyskinetic cilia have abnormal beating, such as bending failure or display a twitching motion. The percentage frequency of dyskinesia was calculated as the number of dyskinetic and immotile cilia per total number of measurements.

### Transmission electron microscopy

The ciliated epithelium for TEM was fixed in 3–4% glutaraldehyde and prepared, imaged and analysed by a researcher unaware of the sample identity, as described previously (Stannard et al., 2010). The percentage of ultrastructural defects was determined by analysis of cilia in cross section. The number of cross sections analysed (typically 300 cilia) was dictated by the size and secondary damage within the fixed sample. Common defects included missing ODAs and IDAs alone or together, truncated ODAs and microtubular disarrangement.

### Electrophysiology

Cells were grown on Costar Snapwell™ Clear permeable supports (Corning Life Sciences, Flintshire, UK) in ALI culture (PneumaCult ALI^TM^, StemCell Technologies, Cambridge, UK) for 30 days. PCD cell lines from successive passages (passages 10 to 18) were utilised in electrophysiology studies. Cells were rinsed twice with growth medium (PneumaCult Ex^TM^, StemCell Technologies, Cambridge, UK) 2 days prior to analysis. Cells were then mounted in Ussing chambers, and 5 ml isotonic physiological salt solution buffer (PSS) was added to either side of the membrane. PSS was composed of NaCl (117 nM), NaHCO_3_ (25 nM), KCl (4.7 nM), MgSO_4_ (1.2 nM), KH_2_PO_4_ (1.2 nM), CaCl_2_ (2.5 nM) and D-glucose (11.0 nM), pH7.4. The solution was maintained at 37°C and continuously bubbled with 21% O_2_ and 5% CO_2_ premixed gas throughout the course of the experiment. The epithelium was clamped at 0 mV and short-circuit current (*I*_sc_) was measured. Every 30 s, a 2 mV pulse was applied to enable calculation of transepithelial resistance (TEER), as described previously (Tagalakis et al., 2018). Spontaneous short-circuit current (*I*_sc_) was measured before addition of the drugs and after addition of each drug to evaluate drug response by observing change in *I*_sc_ (Δ*I*_sc_). Amiloride (10 µM, apical) was used to inhibit ENaC, forskolin (10 µM) and IBMX (10 mM; both bilateral) were used to elevate cAMP and activate CFTR, CFTR_inh_-172 (10 μM, apical) was used to inhibit CFTR, and ouabain (1 mM, basolateral) was used to inhibit Na^+^K^+^ATPase. All drugs were obtained from Sigma-Aldrich.

### Western blotting

Cells in ALI culture on PET transwell inserts (Corning Life Sciences, Flintshire, UK) were lysed using RIPA buffer with protease inhibitor cocktail (Thermo Fisher Scientific, Hemel Hempstead, UK) and incubated on ice for 15 min followed by 15 min centrifugation at 17,000 ***g*** at 4°C. The supernatant was transferred to microfuge tubes (Eppendorf, Stevenage, UK), and protein concentration of the lysate was measured using a bicinchoninic acid (BCA) assay (Thermo Fisher Scientific, Hemel Hempstead, UK). Protein samples were mixed with 6× loading buffer supplemented with denaturing reagent [0.375 M Tris-HCl pH 6.8, 10% (w/v) SDS, 30% (v/v) glycerol, 0.6 M dithiothreitol (DTT), 0.05% (w/v) Bromophenol Blue] and incubated at 37°C for 30 min prior to loading. 17 µg total protein was loaded into each well of NuPAGE 4–12% Bis-Tris protein gels (Thermo Fisher Scientific, Hemel Hempstead, UK). Gel electrophoresis was performed in MOPS buffer for 90 min at 80 mA in an XCell SureLock^TM^ Mini-Cell (Thermo Fisher Scientific, Hemel Hempstead, UK). Proteins were transferred to polyvinylidene difluoride (PVDF) membrane (Millipore, Watford, UK) in transfer buffer [25 mM Tris base, 192 mM glycine, 20% (v/v) methanol] using a Bio-Rad Mini trans-Blot tank (Bio-Rad Laboratories, Watford, UK) for 70 min at 140 V. The membrane was then blocked with 5% dried milk powder in TBS-T (50 mM Tris-base pH 7.5, 150 mM NaCl, 0.2% Tween-20) for 1 h at room temperature. The membrane was incubated with mouse anti-CFTR antibody (AB596, from S. Randell, University of North Carolina, USA; Gentzsch et al., 2007) or rabbit anti-αENaC (SCNN1A) antibody (PA1-920A; Thermo Fisher Scientific, Hemel Hempstead, UK), both diluted 1:1000, or with mouse anti-GAPDH antibody, diluted 1:10,000, in 5% dried milk in TBS-T overnight at 4°C followed by three washes with TBS-T, 10 min each. The membrane was then incubated with rabbit anti-mouse IgG horseradish peroxidase (HRP)-conjugated secondary antibody (1:10,000; Dako, Ely, UK) or goat anti-rabbit IgG HRP-conjugated secondary antibody (1:10000; Dako, Ely, UK) for 1 h in blocking buffer followed by three washes in TBS-T, 10 min each. The blot was developed for imaging using high-sensitivity ECL solution (Pierce ECL plus, Thermo Fisher Scientific, Hemel Hempstead, UK) and imaged using a ChemiDoc™ MP Imaging System (Bio-Rad, Watford, UK).

## Supplementary Material

10.1242/joces.263886_sup1 Supplementary information
