# Supplementary material for: Characterisation of a primary ciliary dyskinesia model generated from BMI1-transduced basal epithelial cells
Source: J Cell Sci. 2025 Oct 31;138(20):jcs263886. doi: 10.1242/jcs.263886 (PMC12633737; doi:10.1242/jcs.263886)
Supplement: Supplementary information [file joces-138-263886-s1.pdf]

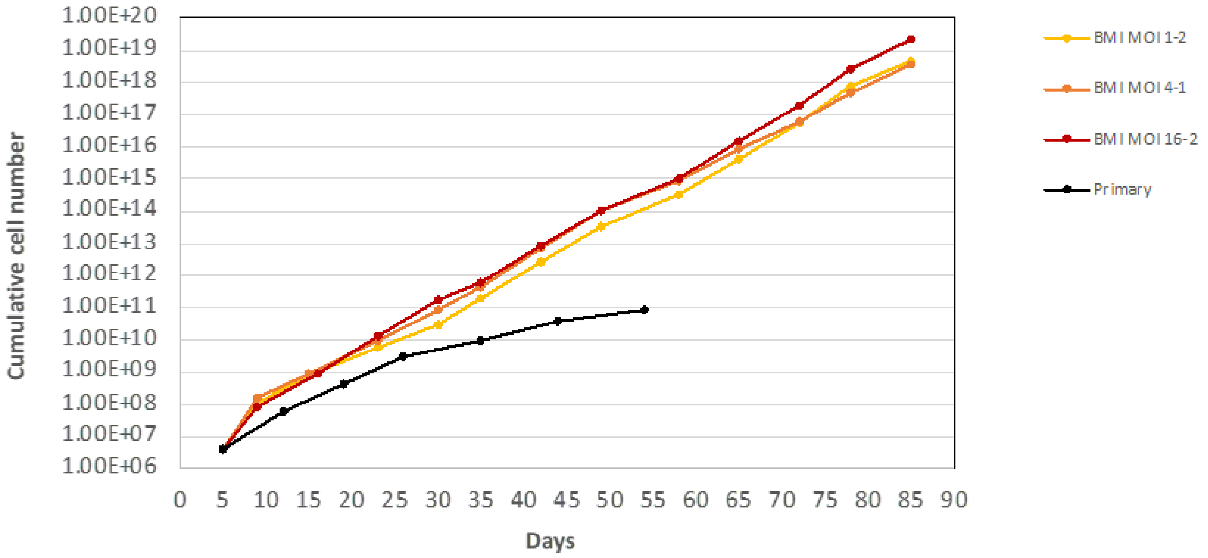

Fig. S1. Growth curve of *BMI1* transduced normal human bronchial epithelial (NHBE) cells Primary NHBE cells were transduced with *BMI1* lentivirus at three MOIs (I, 4 and 16) and were cultured up to 90 days. Growth curves were generated compared to non-transduced primary cells.

Journal of Cell Science • Supplementary information

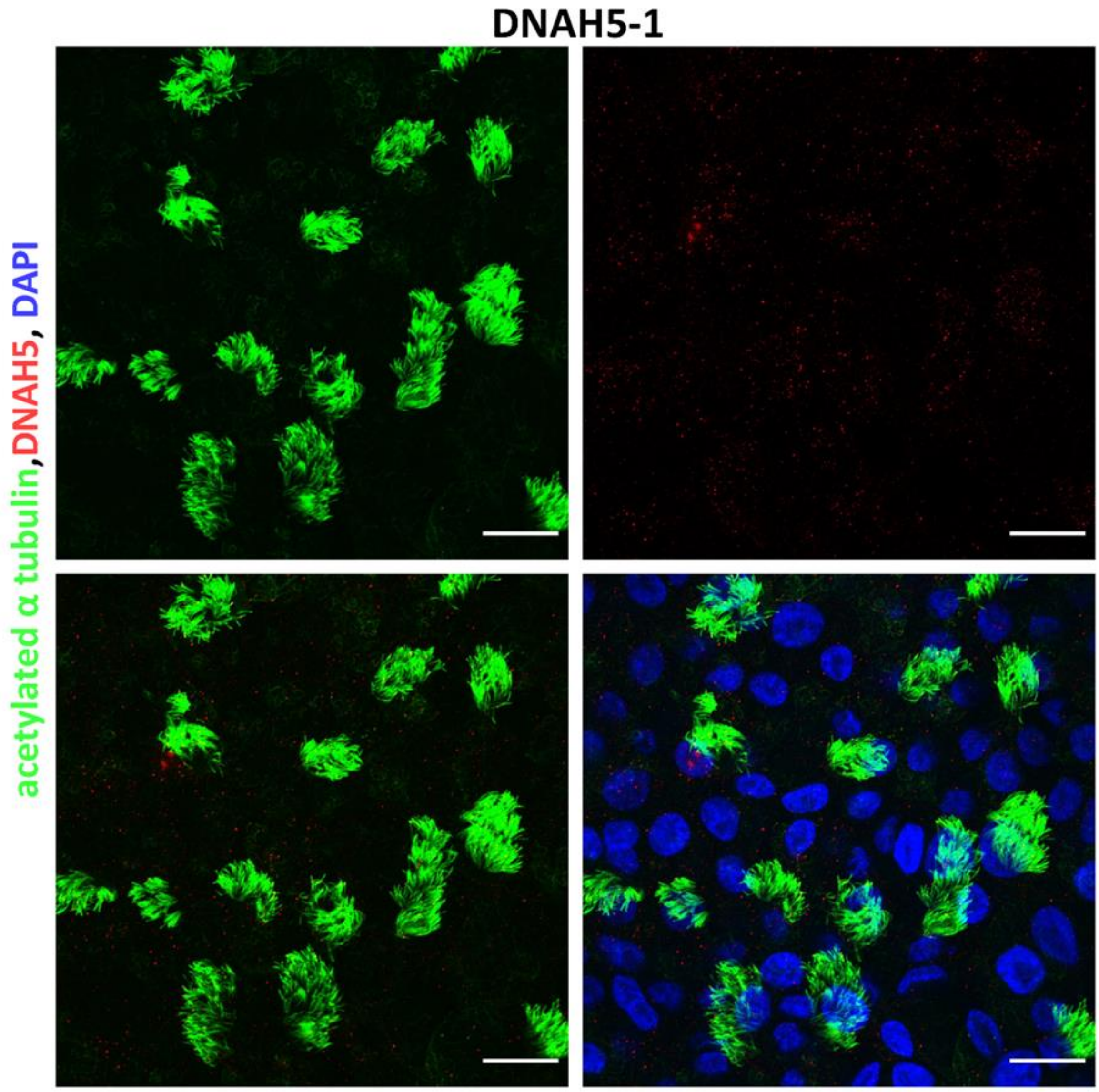

Fig. S2. Characterisation of DNAH5 localisation in *BMI1*-transduced *DNAH5- 1* PCD bronchial airway cell cilia

3D reconstruction of confocal images showing ciliary localisation of acetylated α-tubulin (green) and the absence of DNAH5 (red) in differentiated DNAH5-1 *BMI1* cells of bronchial origin (patient DNAH5-1; p1,10) at day 30 of differentiation. Overlay of both and all channels in bottom panel, DAPI in blue Scale bars represent 20 μm.

Journal of Cell Science • Supplementary information

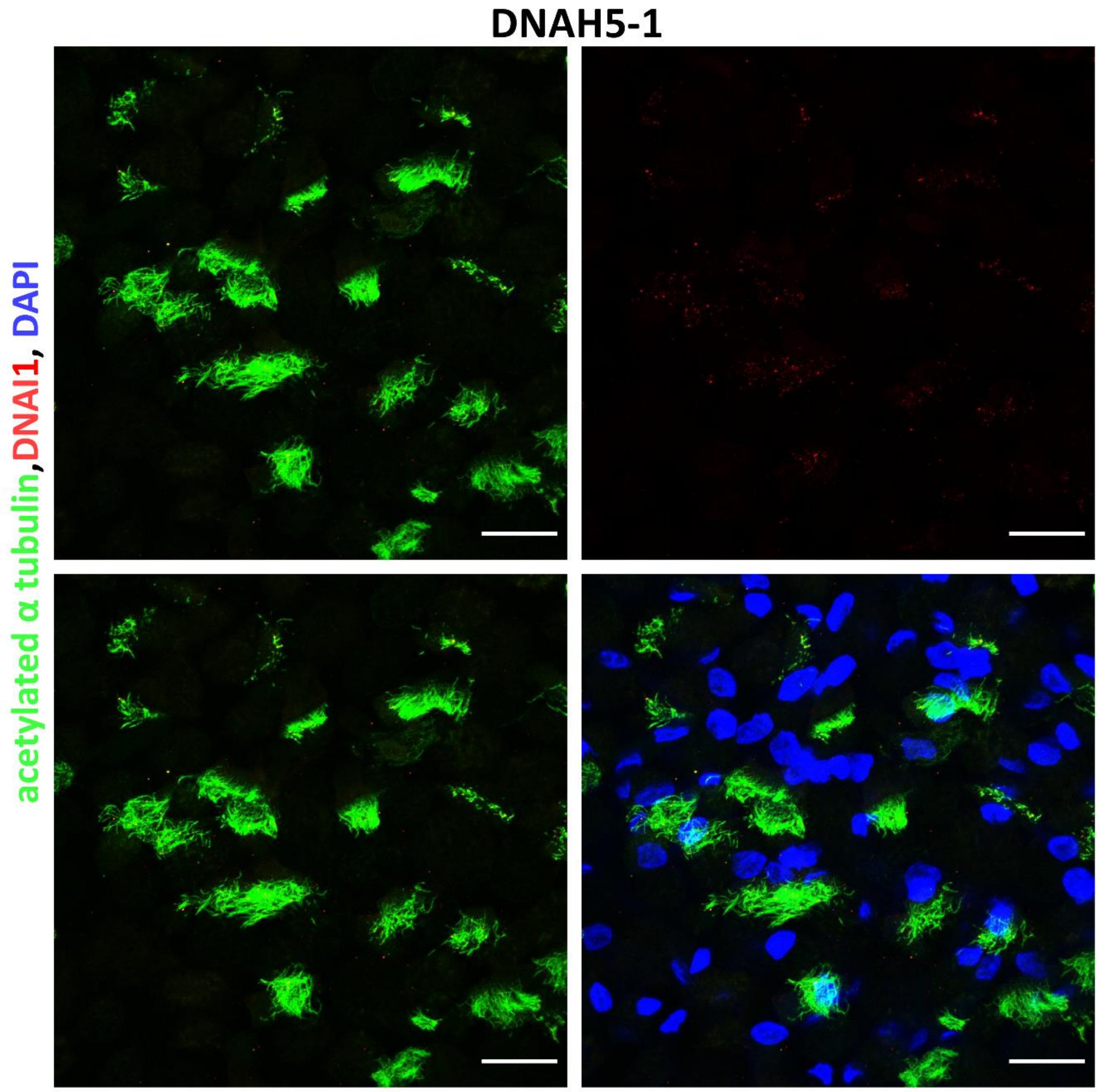

Fig. S3. Characterisation of DNAI1 localisation in *BMI1*-transduced *DNAH5-1* PCD bronchial airway cell cilia

3D reconstruction of confocal images showing ciliary localisation of acetylated α-tubulin (green) and the absence of DNAI1 (red) in differentiated DNAH5-1 *BMI1* cells of bronchial origin (patient DNAH5-1; p1,10) at day 30 of differentiation. Overlay of both and all channels in bottom panel, DAPI in blue Scale bars represent 20 μm.

Journal of Cell Science • Supplementary information

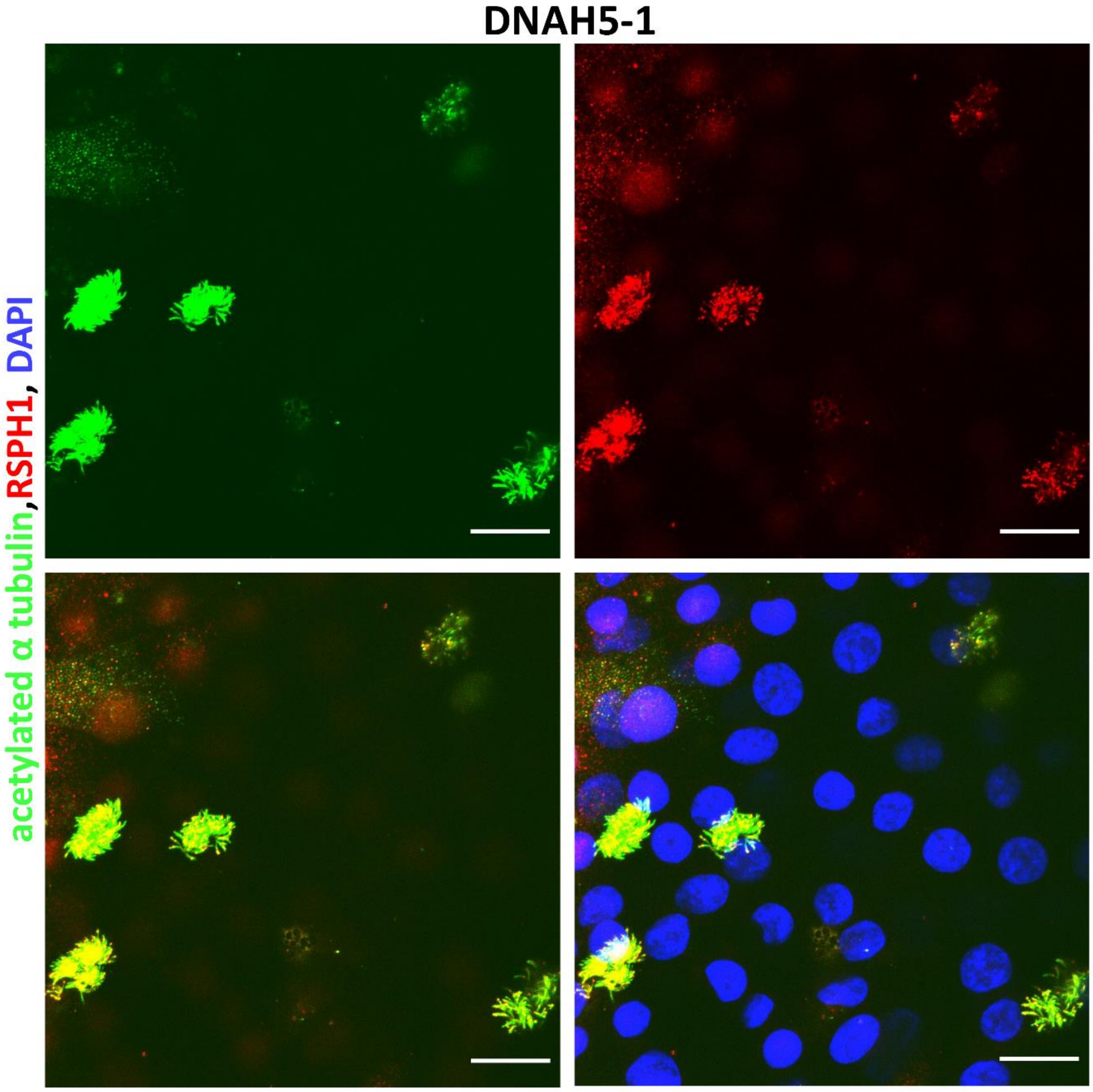

Fig. S4. Characterisation of RSPH1 localisation in *BMI1*-transduced *DNAH5-1* PCD bronchial airway cell cilia

3D reconstruction of confocal images showing ciliary localisation of acetylated α-tubulin (green) and RSPH1(red) in differentiated DNAH5-1 *BMI1* cells of bronchial origin (patient DNAH5-1; p1,10) at day 30 of differentiation. Overlay of both and all channels in bottom panel, DAPI in blue Scale bars represent 20 µm.

Journal of Cell Science • Supplementary information

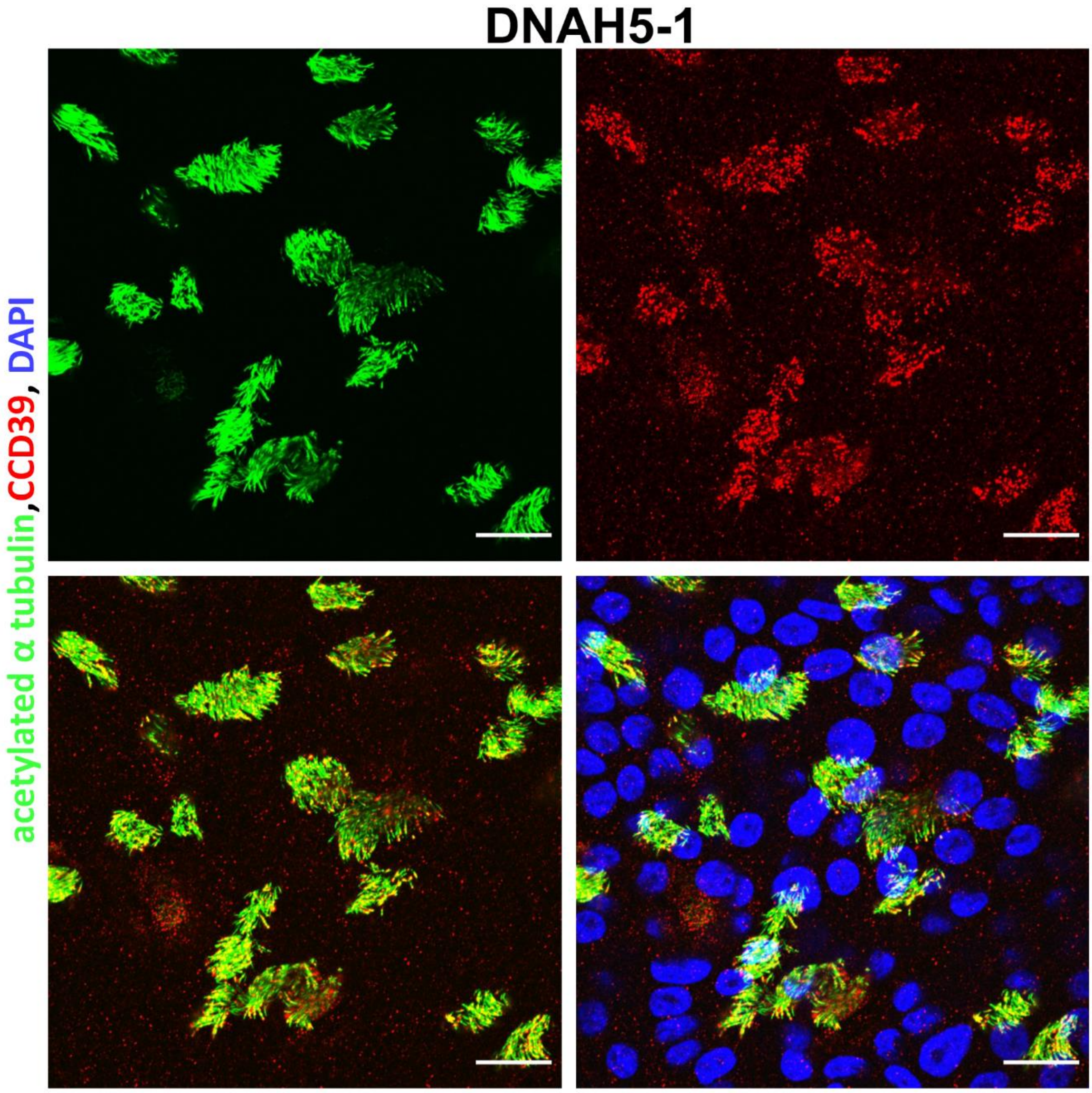

Fig. S5. Characterisation of CCD39 localisation in *BMI1*-transduced *DNAH5-1* PCD bronchial airway cell cilia

3D reconstruction of confocal images showing ciliary localisation of acetylated α-tubulin (green) and CCD39 (red) in differentiated DNAH5-1 *BMI1* cells of bronchial origin (patient DNAH5-1; p1,10) at day 30 of differentiation. Overlay of both and all channels in bottom panel, DAPI in blue Scale bars represent 20 µm.

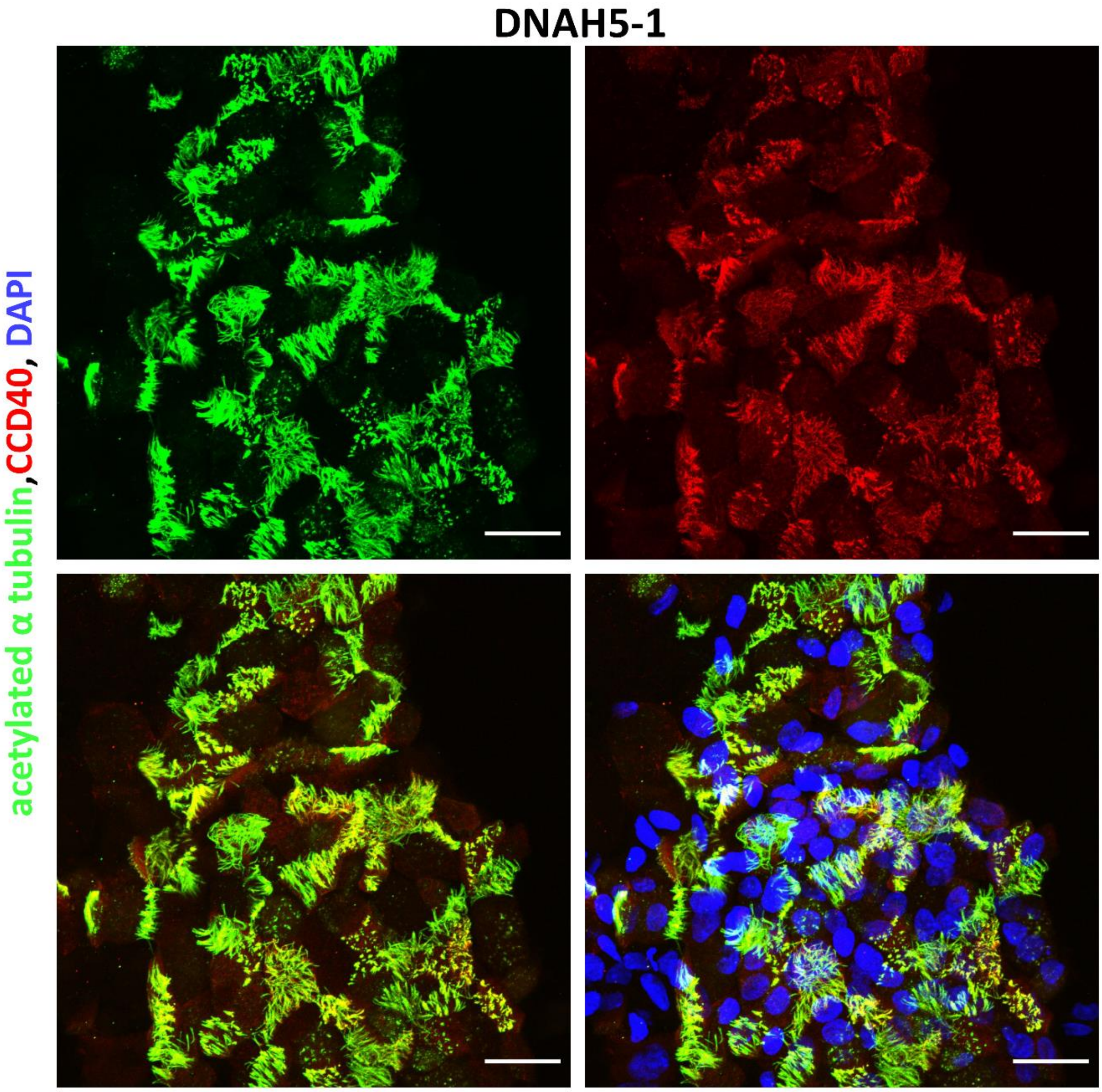

Fig. S6. Characterisation of CCD40 localisation in *BMI1*-transduced *DNAH5-1* PCD bronchial airway cell cilia

3D reconstruction of confocal images showing ciliary localisation of acetylated α-tubulin (green) and CCD40 (red) in differentiated DNAH5-1 *BMI1* cells of bronchial origin (patient DNAH5-1; p1,10) at day 30 of differentiation. Overlay of both and all channels in bottom panel, DAPI in blue Scale bars represent 20 µm.

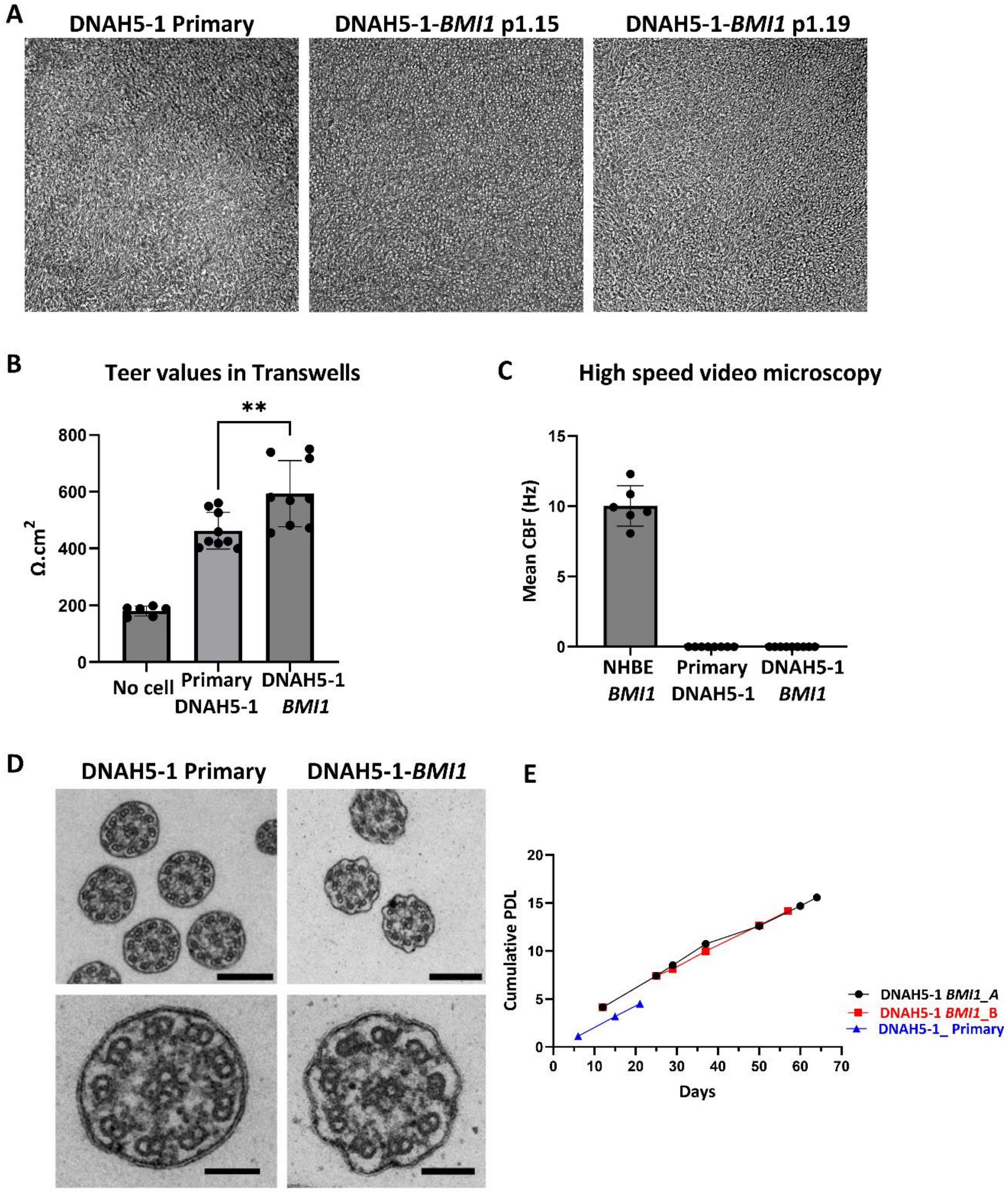

Fig. S7. Analysis of primary DNAH5-1 vs late passage *BMI1* transduced DNAH5-1 airway epithelial cell characteristics.

A. Primary DNAH5-1 cells (p3) and *BMI1*-transduced DNAH5-1 cells (p1,15 and p1,19) grown on transwell membranes at day 60 of ALI cultures. **B.** Epithelial cell resistance of *BMI1*-transduced DNAH5-1 cells (p1,19) were higher than the non-transduced primary DNAH5-1 cells at day 60 of ALI (Mann-Whitney U test, p> 0.01, n=9). **C.** High speed video microscopy recordings show a mean of 10 Hz cilia beating frequency for NHBE cells (p2,13) at day 60 of ALI, indicating functional cilia motility,

while both primary (p3) and *BMI1*-transduced DNAH5-1 cells (p1,19) display immotile cilia (n ≥ 6 fields of view). **D.** TEM analysis of cilia cross section of primary cells from donor DNAH5-1 before *BMI1* transduction (p3) on the left and after *BMI1* transduction (p1,19) on the right, showing missing ODA in both. Scale bars represent 250 nM for the top panel and 100 nM for the lower panel **E.** Growth curve of primary vs *BMI1* transduced DNAH5-1 cells cultured from p1,8 (DNAH5-1 *BMI1*_A) and from p1,12 (DNAH5-1 *BMI1*_B) up to 60 days in culture vs primary DNAH5-1 cells without *BMI1* transduction that stopped growing after p5.

Journal of Cell Science • Supplementary information

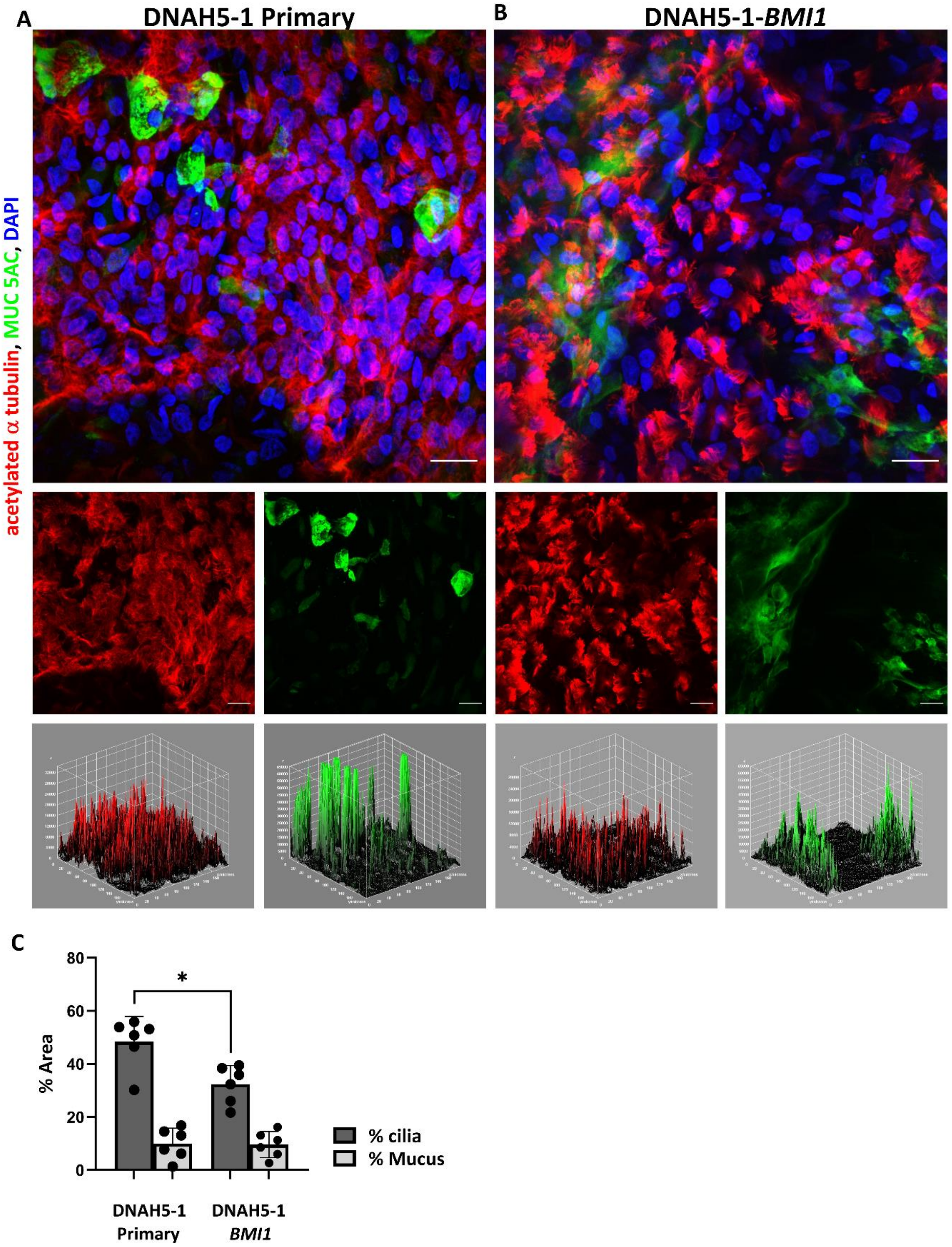

Fig. S8. Characterisation of differentiation in ALI cultures of primary DNAH5- 1 cells and *BMI1* transduced DNAH5-1 cells

3D reconstruction of confocal images stained for acetylated α tubulin (red), MUC5AC (green) and nucleus (DAPI in blue) showing differentiation of basal airway cells into ciliated, mucus-producing cells at day 60 of ALI **A.** Primary DNAH5-1 cells before transduction (p3) and **B.** DNAH5 *BMI1* cells from donor DNAH5-1 (p1,19). Scale bars represent 20 µm. **C**. Quantification of cilia % area and MUC5AC % area in both primary DNAH5-1 and DNAH5-1 *BMI1* airway epithelial cells in ALI culture at Day 60 of differentiation from 3D reconstruction of confocal images, (n=6 fields per group,*$p < 0.05$, Mann-Whitney U test).

Journal of Cell Science • Supplementary information

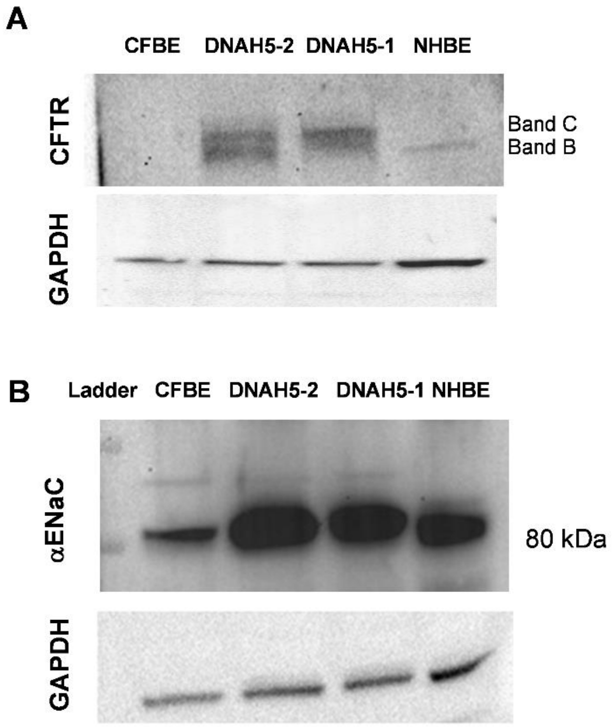

Fig. S9. Immunoblotting for αENaC and CFTR.

Protein samples from PCD-*BMI1* epithelial cells (DNAH5-1 and DNAH5-2), CF-*BMI1* bronchial epithelial cells (CFBE) and normal human bronchial epithelial cells-*BMI1* (NHBE) were analysed by electrophoretic separation and immunoblotting for CFTR (A) and αENaC (B). A) The double bands for CFTR are characteristic of the lower molecular weight, core glycosylated protein (B form) and the slower migrating, complex glycosylated protein (C form). B) αENaC staining reveals the ~80 kDa protein. To ensure similar levels of protein, samples were also stained for the housekeeping gene, GAPDH.

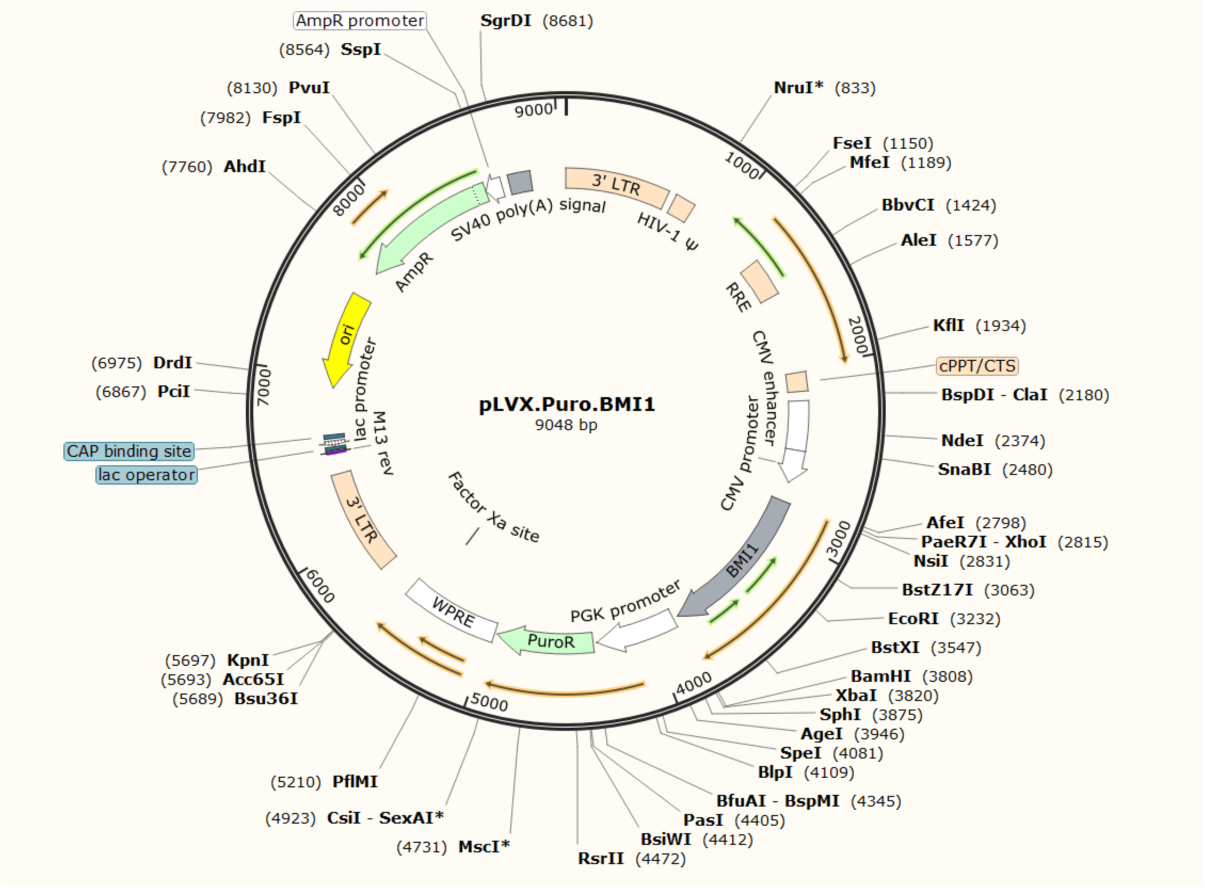

Fig. S10. pLVX.Puro.BMI1 plasmid map

Journal of Cell Science • Supplementary information

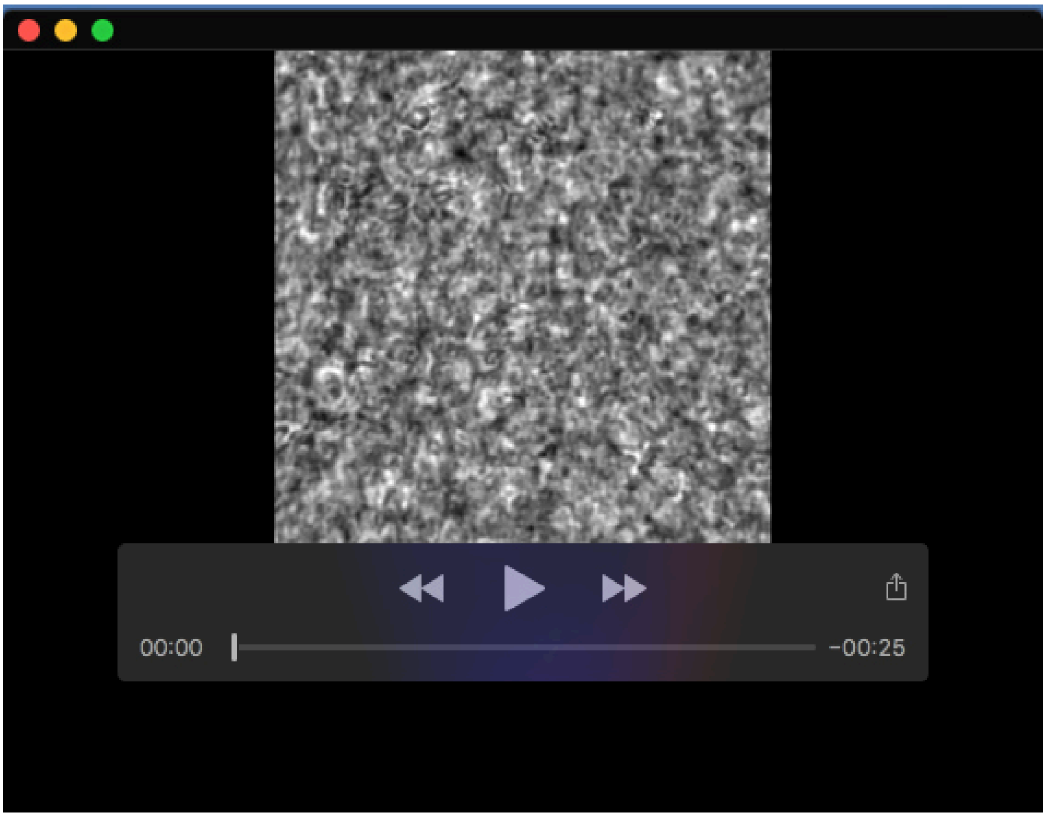

Movie 1. High speed video microscopy of NHBE-BMI1 cells on transwells at day 35 of ALI culture displaying motile cilia.

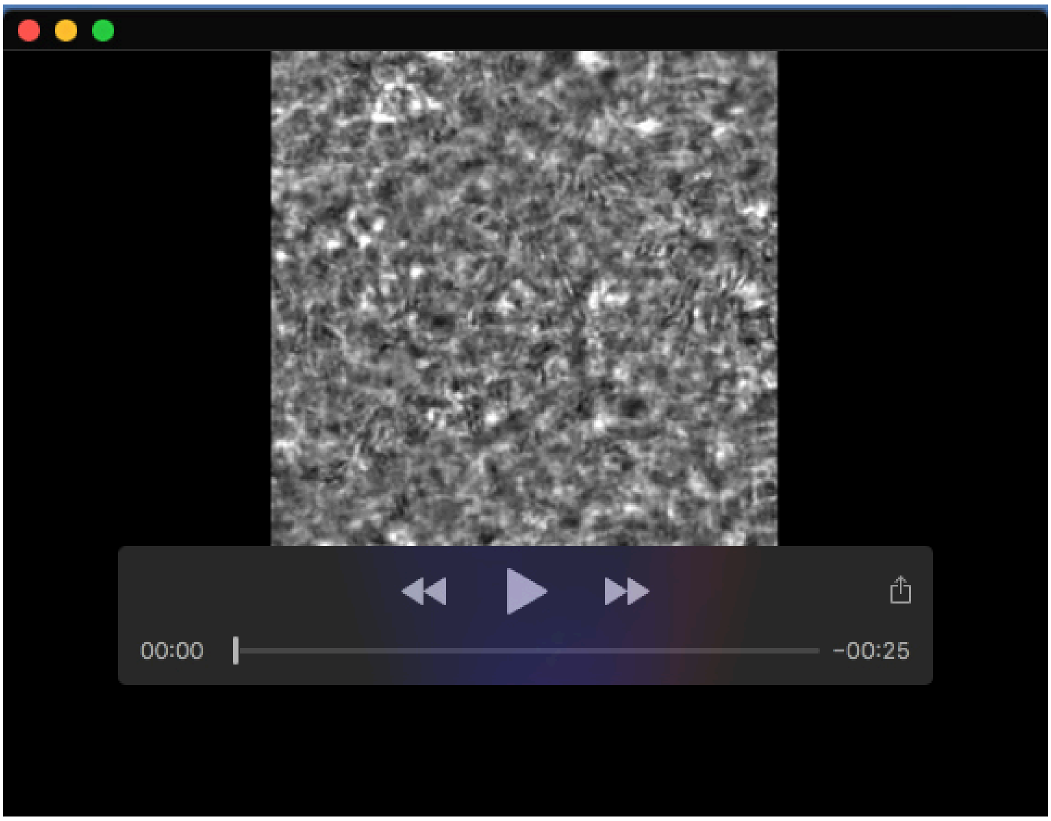

Movie 2. High speed video microscopy of NHBE-BMI1 cells on transwells at day 60 of ALI culture displaying motile cilia.

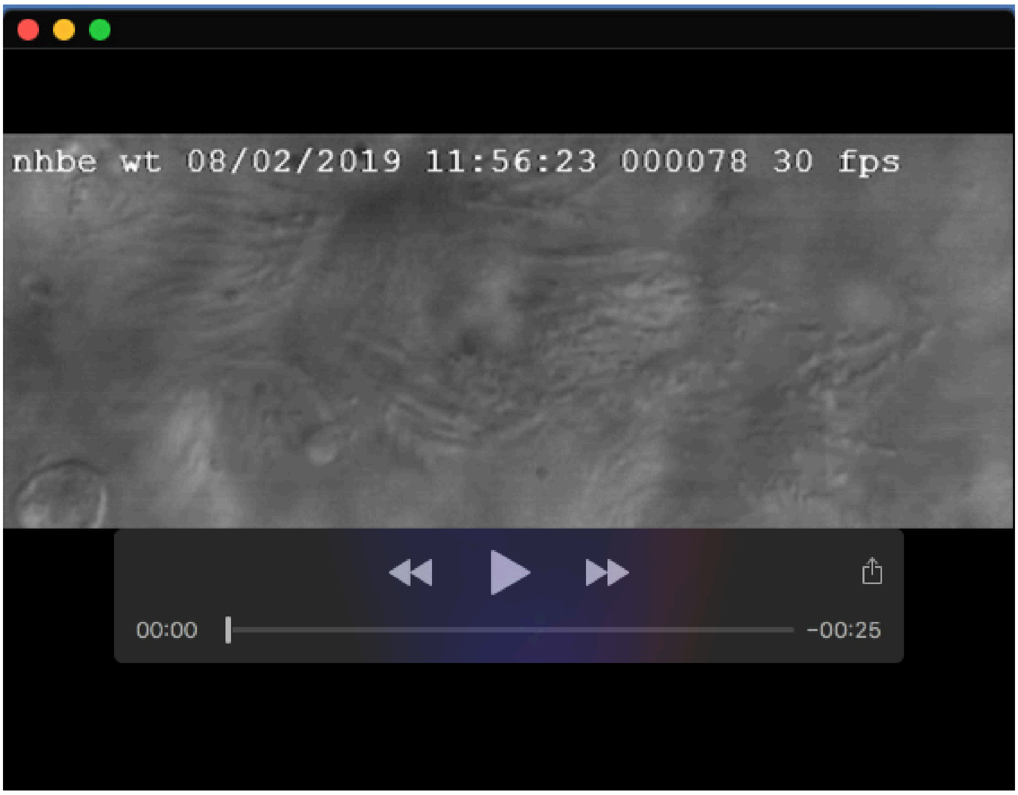

Movie 3. Light microscopy recordings of NHBE-BMI1 cells on a slide at day 60 of ALI culture displaying motile cilia from top view.

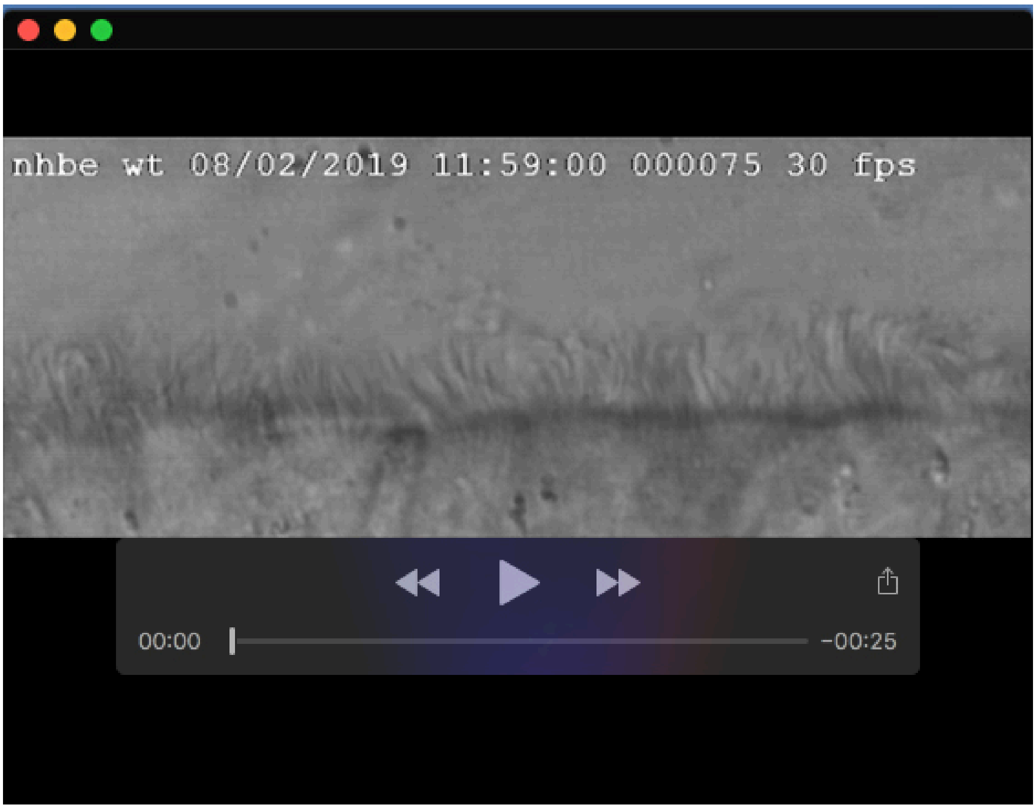

Movie 4. Light microscopy recordings of NHBE-BMI1 cells on a slide at day 60 of ALI culture displaying motile cilia from side view.

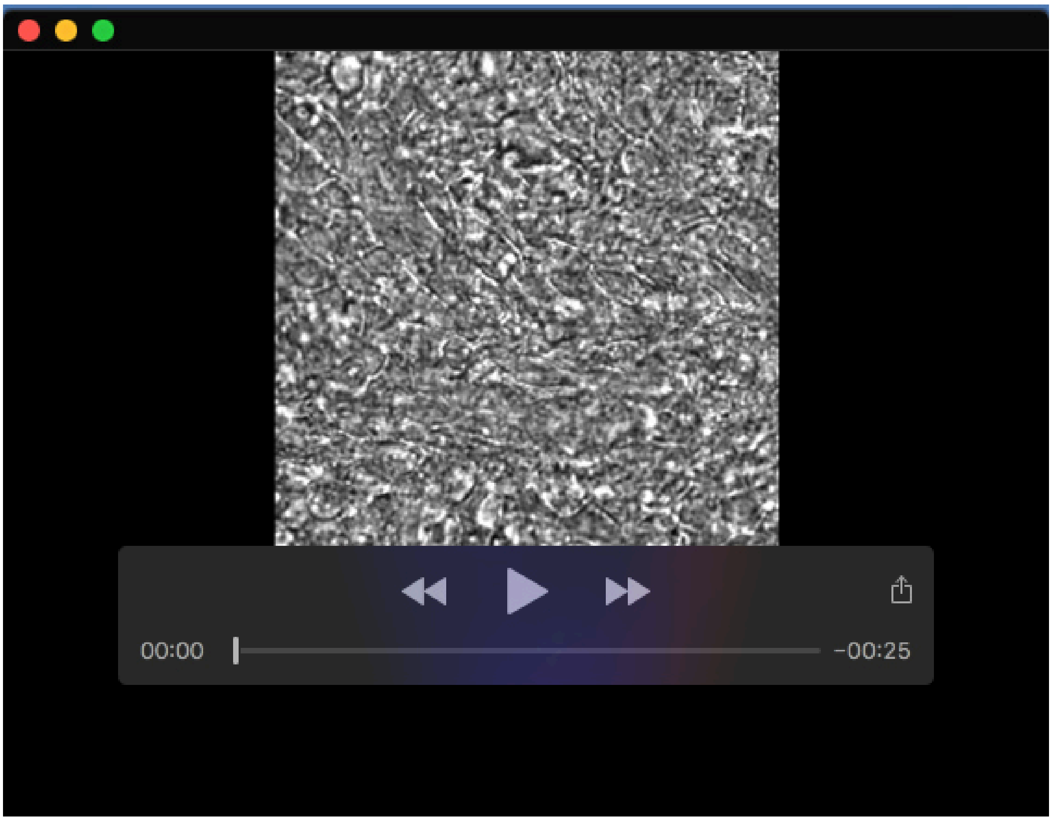

Movie 5. High speed video microscopy of DNAH5-2 -BMI1 cells on transwells at day 35 of ALI culture displaying immotile cilia.

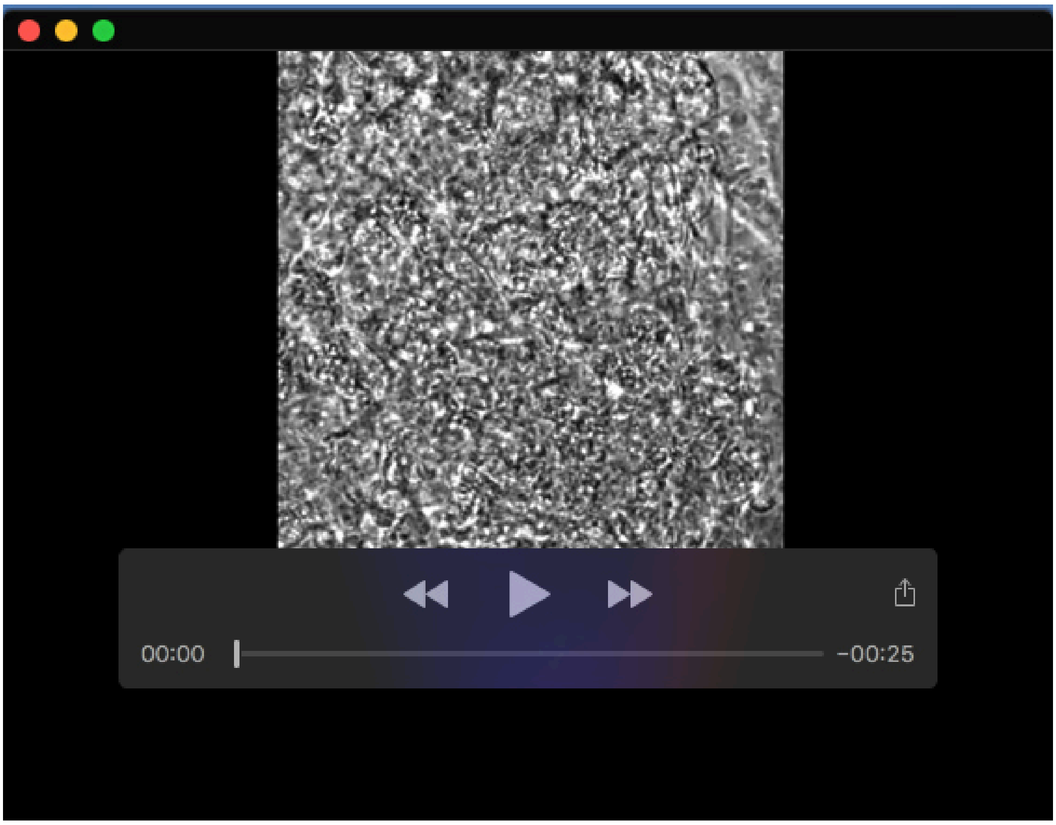

Movie 6. High speed video microscopy of DNAH5-2 -BMI1 cells on transwells at day 60 of ALI culture displaying immotile cilia.

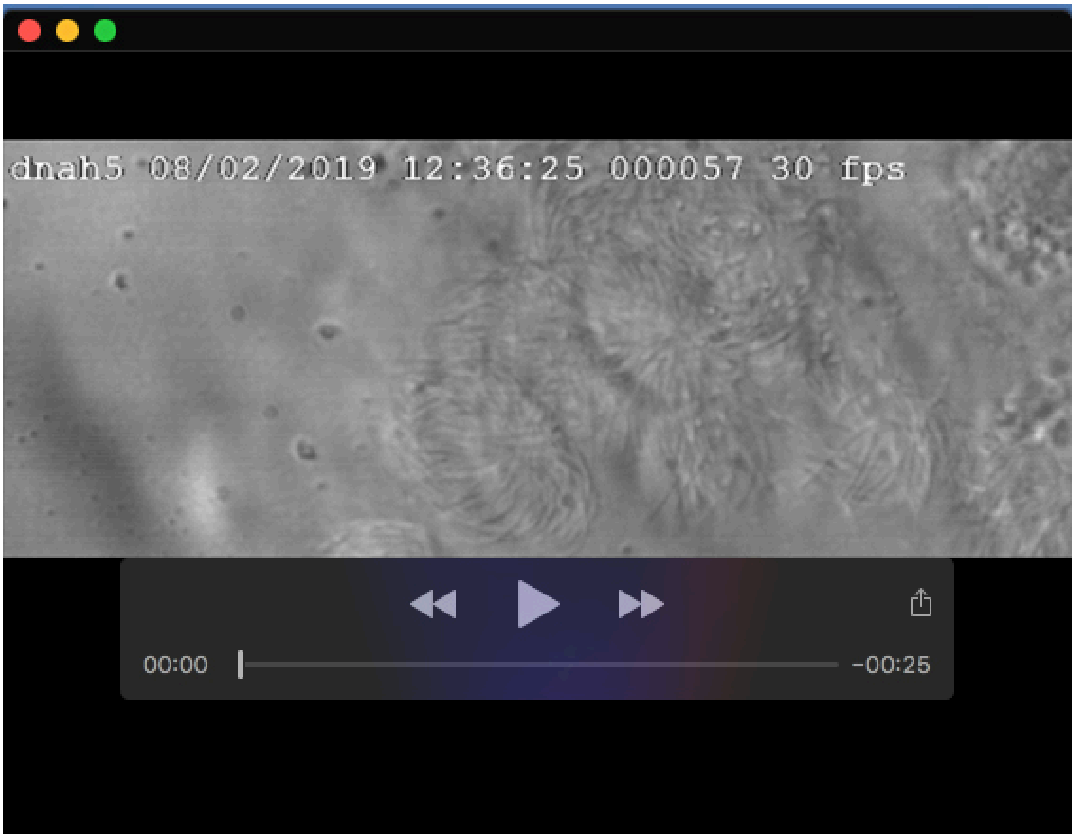

Movie 7. Light microscopy recordings of DNAH5-2-BMI1 cells on a slide at day 60 of ALI culture displaying mostly static cilia from top view.

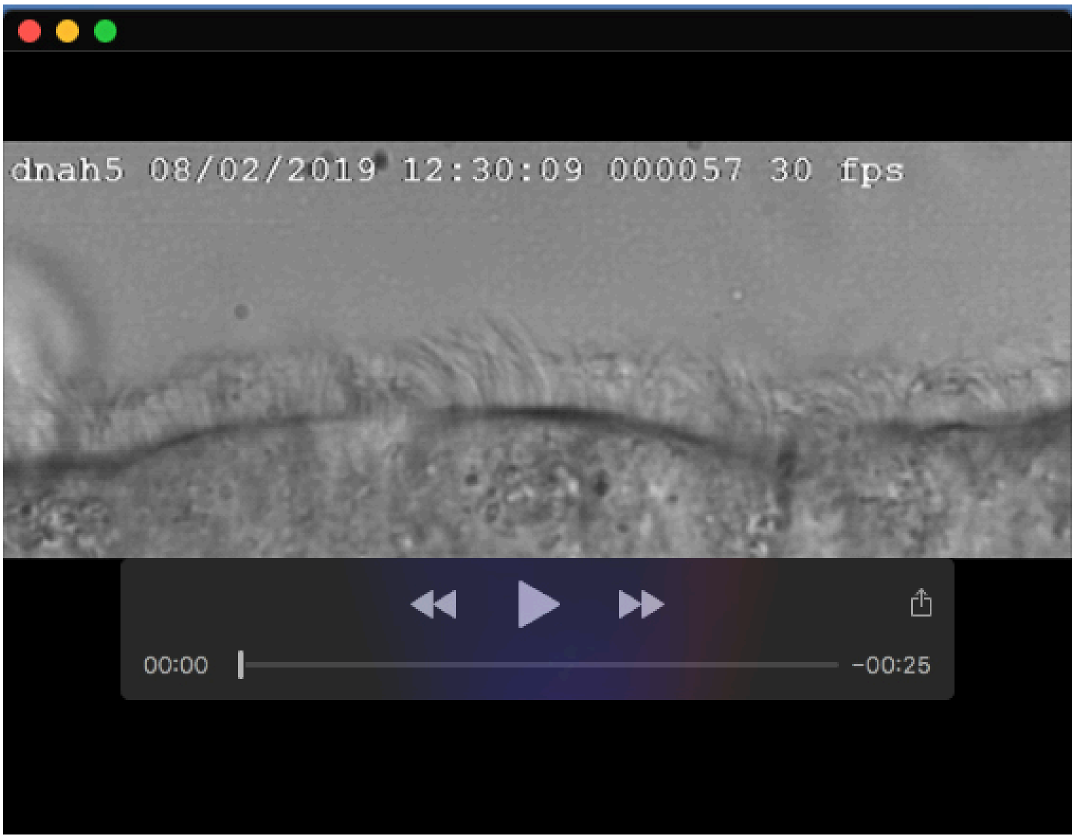

Movie 8. Light microscopy recordings of DNAH5-2-*BMI1* cells on a slide at day 60 of ALI culture displaying mostly static cilia from side view.

Journal of Cell Science • Supplementary information
